# Bioinspired Mechanosensitive Organic Artificial Neuron With Programmable Excitatory and Inhibitory Responses via Nonlinear Mechano‐Electrochemical Coupling

**DOI:** 10.1002/adma.75027

**Published:** 2026-09-17

**Authors:** Rassen Boukraa, Pietro Belleri, Zsolt M. Kovács‐Vajna, Paschalis Gkoupidenis, Fabrizio Torricelli

**Affiliations:** ^1^ Department of Information Engineering University of Brescia Brescia Italy; ^2^ Max Planck Institute for Polymer Research Mainz Germany; ^3^ Department of Electrical and Computer Engineering North Carolina State University Raleigh USA; ^4^ Department of Physics North Carolina State University Raleigh USA

**Keywords:** multimodal neuromorphics, organic artificial neuron, organic electrochemical transistors, spiking neurons

## Abstract

Neuromorphic systems capable of directly interfacing with the physical world are essential for embodied intelligence, wearable bioelectronics, and adaptive human‐machine interaction. However, most artificial neurons process mechanical and chemical stimuli through external sensors and peripheral circuitry, rather than encoding multimodal information at the neuron‐level, limiting integration and functional coupling between stimuli and excitability. Here we report a flexible organic artificial neuron (OAN) that couples mechanical deformation and ionic environment to spiking dynamics through nonlinear mechano‐electrochemical interactions. The OAN, fabricated by additive manufacturing on flexible substrates, integrates an organic electrochemical nonlinear element exhibiting S‐shaped negative differential resistance (S‐NDR) with a nonlinear active load to generate low‐voltage oscillatory activity. Mechanical bending within the elastic regime and variations in electrolyte ion concentration reconfigure the relative position between the S‐NDR characteristic and the active‐load line in the current‐voltage plane, enabling deterministic transitions between quiescent and oscillatory states. The proposed OAN shows mechanically triggered firing, mechanically induced silencing, and chemically controlled reactivation. Numerical simulations quantitatively reproduce the nonlinear OAN operation and spiking behavior, providing predictive design guidelines. These findings establish a device‐level framework for multimodal organic neurons in which mechanical and ionic signals are directly encoded in device physics, opening avenues toward biointegrated neuromorphic systems.

## Graphical abstract

Organic artificial neurons couple mechanical deformation and ionic environments through nonlinear mechano‐electrochemical dynamics. Mechanical strain and electrolyte concentration reshape their nonlinear electrical characteristics, programming excitatory or inhibitory spiking responses that mimic mechanosensitive biological neurons and enable multimodal neuromorphic bioelectronics.

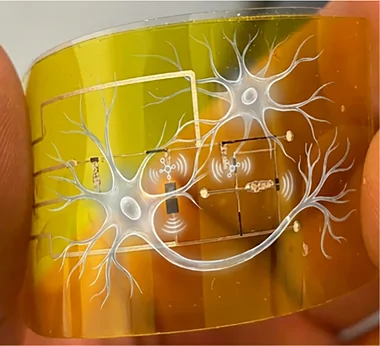

## 1 Introduction

Neuromorphic electronics aim to emulate the brain's ability to efficiently process a wide range of biological and environmental signals. By mimicking key elements of the brain, such as neurons and synapses, these systems enable bio‐inspired sensing and processing, unlocking new possibilities in fields such as neuromorphic and edge computing, healthcare and medical devices, wearables, artificial sense, intelligent agents, robotics and prosthetics [1, 2, 3, 4]. Among the various materials approaches for neuromorphic electronics, soft organic materials are particularly noteworthy due to their responsiveness to a broad spectrum of signals—including electrical, chemical, mechanical, optical, and thermal [4, 5, 6, 7, 8]. This wide‐ranging sensitivity enables them to create multimodal interactions with the external environment [9, 10, 11, 12, 13]. This multimodality represents a significant recent development in the field of neuromorphic electronics. It plays a crucial role in enabling embodied intelligence, facilitating the collection, processing, computation, and delivery of biologically and environmentally relevant sensorimotor signals [14, 15, 16, 17, 18, 19].

In recent years, the fundamental components of the nervous system have been successfully replicated using organic electrochemical transistors (OECTs) [20, 21, 22, 23, 24, 25], leading to the development of low‐voltage artificial synapses and bio‐realistic artificial neurons [26, 27, 28]. For instance, organic electrochemical synapses enable a wide range of non‐volatile, linearly tunable conductance for the realization of small‐scale artificial neural networks [29], circuits for on‐chip diagnostics [30], and intelligent systems [31, 32]. Moreover, electrochemical organic artificial neurons (OANs) are adept at replicating biological phenomena found in natural neurons [28, 33]. They respond effectively to key biochemical signals, including alkaline ions, neurotransmitters, and biomolecules [34, 35, 36, 37, 38]. Nonlinear electrochemical phenomena play a central role in the operation of OANs [35, 38]. For example, the S‐shaped negative differential resistance (S‐NDR) of OANs and how it is probed define with high precision the basic neuronal functions such as excitability and spiking modes [35].

More broadly, nonlinear device characteristics such as negative differential resistance (NDR) and negative differential transconductance (NDT) have emerged as powerful physical primitives for compact spiking neurons. Tunable Gaussian heterojunction transistors have been used to implement spiking neuron circuits through electrostatic control of nonlinear transfer characteristics [39], while metal oxide/small‐molecule heterojunction N‐shape switching transistors have recently been exploited for random number generation and spiking neuron operation [40]. These studies highlight the importance of nonlinear circuit elements for spike generation. Here, we extend this concept to a flexible organic electrochemical platform in which the S‐NDR/load‐line configuration is not only electrically biased but directly reshaped by mechanical strain and electrochemically tuned by ion concentration, enabling programmable excitatory and inhibitory responses.

Despite the recent advancements in OANs, the development of multimodal neurons requires a more comprehensive framework of nonlinear phenomena, which encompasses arbitrary signals and their associations. A prime example from the biological world is mechanosensitive ion channels. These channels underpin the function of mechanosensitive neurons, as depicted in Figure 1a. Found in various tissues including the skin, gut, heart, and retina, these neurons are specifically activated by mechanical pressure, triggering neuronal excitation [41, 42, 43]. In this example, there is a coupling between the mechanical deformation of the biological membrane and the conductance of mechanosensitive ion channels (i.e., ion conductance through the channel). This interaction specifically involves the transmembrane ion transport through the mechanosensitive ion channels. Although artificial neurons translating pressure or mechanical deformation into spiking activity exist [44, 45, 46, 47], current approaches rely on pressure sensors or mechanical transducers that are interfaced externally with the neural circuit, translating mechanical input into an electrical signal that is then processed by a separate neuron stage. As a result, the mechanical stimulus modulates the neuron input but does not reshape its intrinsic nonlinear dynamics, leaving excitability, spiking threshold, and ionic coupling fundamentally decoupled from the mechanical domain. Linking mechano‐electrochemical stimuli and the nonlinear dynamics of artificial neurons has not yet been established. This feature is essential for creating mechanosensitive artificial neurons in which excitability, spiking and ion dynamics are directly incorporated into the signaling process. Establishing this link would provide the foundation for artificial systems capable of mimicking biological mechanotransduction, opening pathways toward adaptive neuromorphic devices responsive to both mechanical and ionic stimuli.

**FIGURE 1:**
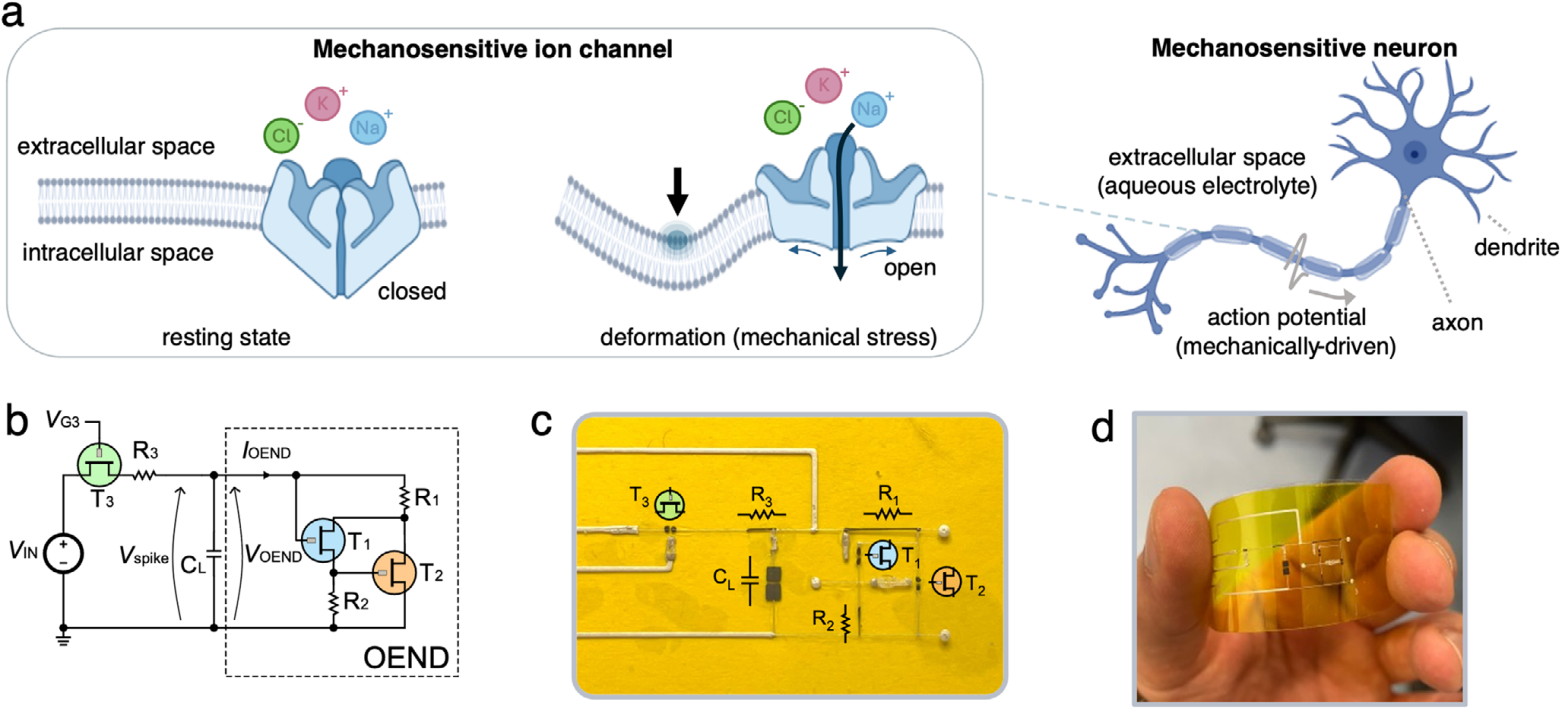
Mechanosensitive OAN. (a) Biological mechanosensitive neuron and its underlying transduction mechanism: mechanical deformation of the cell membrane opens stretch‐activated ion channels, enabling selective ion influx and triggering neuronal excitation. (b) Circuit schematic of the OAN, comprising the OEND connected to the nonlinear load network (*T*_3_ and *R*_3_) and the load capacitor *C*_L_. (c) Top‐view photograph of a fully printed OAN monolithically integrated on polyimide substrate, with circuit components annotated alongside their physical counterparts. (d) Photograph of the OAN under applied mechanical bending, illustrating the flexible substrate deformation that constitutes the mechanical stimulus.

In this work, we address this challenge by demonstrating a monolithically integrated OAN on a flexible substrate in which nonlinear mechano‐electrochemical coupling directly governs multimodal neural functionality. By establishing a quantitative link between mechanical deformation, ionic transport, and the OAN S‐shaped negative differential resistance (S‐NDR), we show that substrate bending and electrochemical stimuli—applied independently or concurrently—shift the S‐NDR characteristic continuously across the current‐voltage plane, providing on‐demand control over excitatory and inhibitory regimes. This modulation manifests as dynamic transitions between quiescent and bursting states through the controlled initiation or suppression of action potentials. Quantitative agreement between experiments and numerical simulations of the mechano‐electrochemical nonlinear dynamics validates a predictive modeling framework for this device class. These results establish a rigorous device‐ and circuit‐level foundation for multimodal artificial neurons, opening new pathways for embodied intelligence architectures and event‐driven sensorimotor systems.

## 2 Results and Discussion

The OAN is configured by integrating an organic electrochemical nonlinear device (OEND) with a nonlinear network and a direct‐current (DC) voltage source (*V*_IN_), as illustrated in Figure 1b. The OEND consists of two OECTs, designated *T*_1_ and *T*_2_, along with two resistors, *R*_1_ and *R*_2_. The nonlinear network comprises an OECT named *T*_3_ connected in series with a resistor *R*_3_, and a capacitor *C*_L_ is connected in parallel to the OEND. The OECT *T*_1_ and *T*_3_ are p‐type depletion‐mode transistors (positive threshold) based on the organic mixed ionic–electronic conductor poly(3,4‐ethylenedioxythiophene) doped with poly(styrene sulfonate) (PEDOT:PSS), while *T*_2_ is a p‐type accumulation‐mode transistor based on PEDOT:PSS co‐formulated with diethylenetriamine (DETA), a de‐doping agent [48]. All the components of the OAN, viz. OECTs, resistors and capacitor, are monolithically integrated on a plastic substrate by using a micro‐dispensing method.

Micro‐dispensing is an additive manufacturing technique that enables material deposition across a wide viscosity range—from a few centipoises to more than 10^5^ cPs—without the need for masks, etching, or vacuum processing, making it particularly suited for the rapid prototyping of heterogeneous device architectures on flexible substrates [49, 50]. The main steps of the fabrication process are schematically depicted in Figure S1. In the first step, we deposited a low‐temperature cure silver conductor to define the electrical interconnects between components. In the second step, we micro‐dispensed a carbon conductor defining the geometries of the source and drain electrodes of the OECTs (*T*_1_, *T*_2_, and *T*_3_), of the resistors (*R*_1_, *R*_2_, and *R*_3_), and of the *C*_L_ capacitor electrodes. The carbon conductor is ion impermeable and operates as a polarizable material that suppresses redox reactions with the electrolyte. Importantly, transitioning between materials—such as from silver to carbon—requires no cleaning or purging steps and generates no material waste, underscoring the process simplicity and sustainability. Subsequently, we deposited the PEDOT:PSS and PEDOT:PSS:DETA polymers defining the OECT channels. PEDOT:PSS was also deposited on the carbon electrodes of the capacitor to obtain a volumetric capacitance. The gate of the OECTs is a nonpolarizable electrode obtained by micro‐dispensing silver/silver‐chloride on the side of the channel. The use of a nonpolarizable electrode provides very efficient gating while minimizing the gate area [51]. Finally, a layer of polystyrene sulfonate sodium (PSS:Na) is deposited on top of the gate and the corresponding channel of each OECT. This polyelectrolyte hydrated with sodium‐chloride aqueous solution provides a solid‐state ion reservoir (SSIR) to ensure an efficient ionic coupling between the gate and the channel. Moreover, the SSIR was deposited on the two capacitor electrodes to form a volumetric electrolytic capacitor (Figure S2). Measured transfer characteristics (*I*_D_–*V*_G_) and output characteristics (*I*_D_–*V*_D_) of the PEDOT:PSS and PEDOT:PSS:DETA OECTs are presented in Figures S3 and S4, respectively. Further details on the OAN fabrication are provided in the Section 4. Figure 1c shows a photograph (top view) of an OAN monolithically integrated on a polyimide substrate and, for the sake of clarity, the various components are also sketched. Figure 1d shows a fabricated OAN under applied mechanical bending.

Measured current–voltage (*I*_OEND_–*V*_OEND_) characteristics (symbols) of an OEND operated in voltage mode (*V*_OEND_ applied and *I*_OEND_ measured), and operated in current mode (*I*_OEND_ applied and *V*_OEND_ measured) are displayed in Figure 2a,b, respectively. A hysteretic *I*_OEND_–*V*_OEND_ characteristic is obtained when the OEND is operated in voltage mode. According to our previous work [35], this is inherently due to the different threshold voltages of the depletion‐mode OECT *T*_1_ and accumulation‐mode OECT *T*_2_ and the positive feedback of *R*_1_, which give rise to voltage‐dependent conductive paths through the branches *R*_1_–*T*_1_–*R*_2_ or *R*_1_–*T*_2_. During the forward voltage sweep of *V*_OEND_ from 0 V to a value lower than a turn‐on voltage (*V*_ON_ ∼ 1.1 V in Figure 2a), the current *I*_OEND_ flows through the *R*_1_–*T*_1_–*R*_2_ path because *T*_1_ is a depletion‐mode (normally‐on) p‐type OECT and *T*_2_ is an accumulation‐mode (normally‐off) p‐type OECT. *T*_1_ operates in the linear region, which results in a small channel resistance (R*T*_1_) and a small source‐drain voltage *V*_SD1_ = *RT*_1_ × *I*_OEND_. This results in a slope of the *I*_OEND_–*V*_OEND_ characteristic equal to d*I*_OEND_/d*V*_OEND_ = 1/(*R*_1_ + *RT*_1_ + *R*_2_). As *V*_OEND_ increases, the source‐drain voltage of *T*_1_ (*V*_SD1_) also rises. Due to the circuit configuration, *V*_SD1_ = *V*_SG2_, and once *V*_SD1_> |*V*_TH2_|, *T*_2_ turns on, its channel resistance (*RT*_2_) becomes much smaller than *R*_2_, and the current flows primarily through the *R*_1_–*T*_2_ branch, causing a sharp increase in current. This nonlinear current rise leads to a significant voltage drop across *R*_1_. Since V*R*_1_ = *V*_GS1_, the overdrive voltage of *T*_1_ decreases, and eventually, when *V*_GS1_> *V*_TH1_, *T*_1_ turns off. The voltage needed to turn *T*_2_ on is called *V*_ON_. As *V*_OEND_ continues to increase beyond *V*_ON_, *I*_OEND_ increases linearly with a slope of d*I*_OEND_/d*V*_OEND_ = 1/(*R*_1_ + *RT*_2_). When *V*_OEND_ is reduced, *I*_OEND_ decreases linearly, leading to a decrease in *V*_GS1_ = *R*_1_ × *I*_OEND_. Once *V*_GS1_< *V*_TH1_, *T*_1_ switches on in the linear region, where R*T*_1_ is small, and *V*_SD1_ = R*T*_1_ × *I*_OEND_ becomes small as well. Since *V*_SD1_ = *V*_SG2_, *T*_2_ turns off when *V*_SG2_< |*V*_TH2_|, resulting in a sharp decrease in the OEND current. The voltage required to turn off *T*_2_ is referred to as *V*_OFF_. Thus, the OEND exhibits hysteretic behavior, with nonlinear current switching between the *R*_1_–*T*_1_–*R*_2_ and *R*_1_–*T*_2_ branches during forward and reverse voltage sweeps. The same branch switching mechanism gives rise to the S‐NDR characteristic when the OEND is probed in current mode, as discussed below.

**FIGURE 2:**
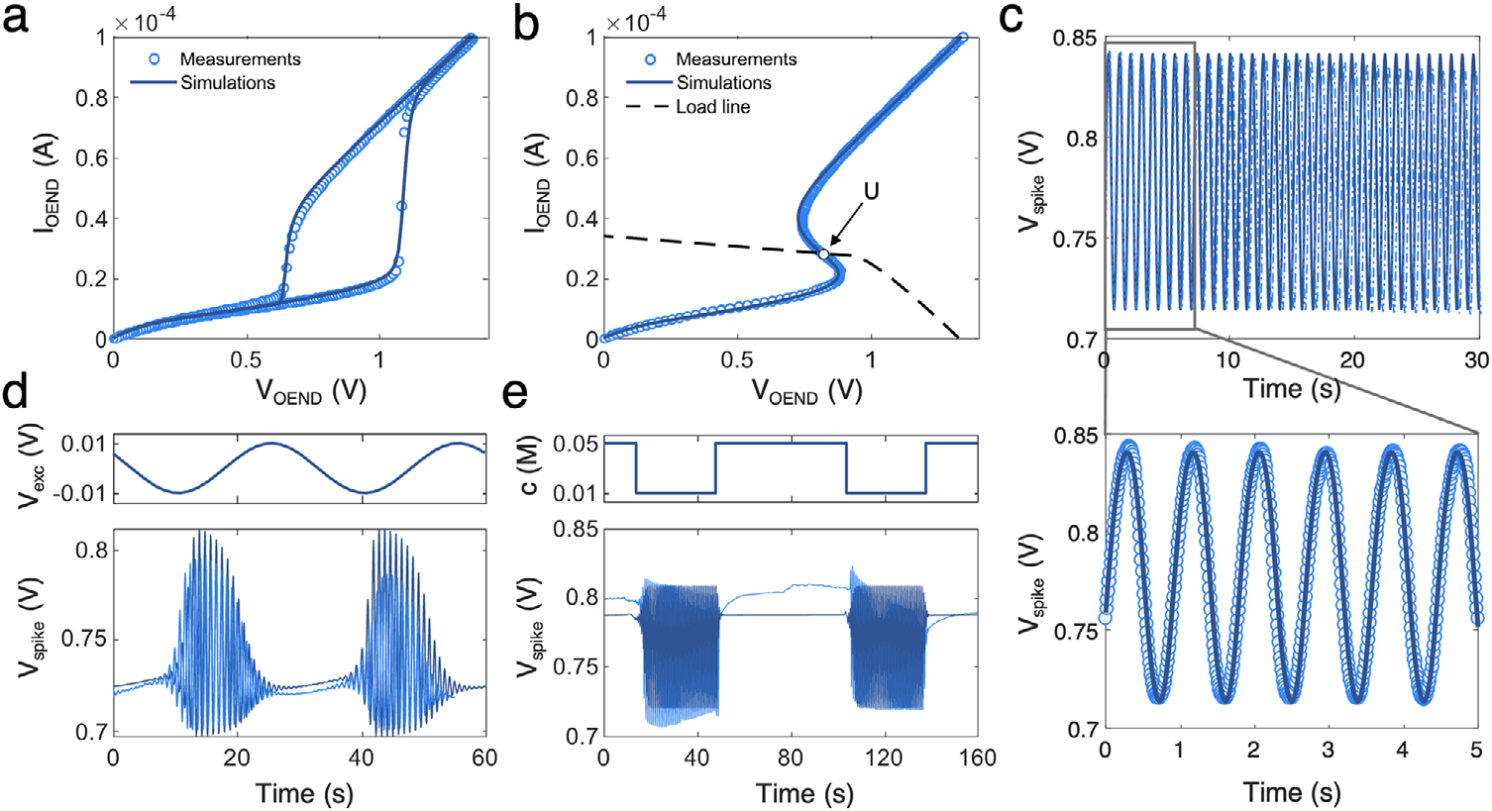
Electrical characterization and neuronal dynamics of the OAN. (a) Measured (symbols) and simulated (full line) electrical characteristic of the OEND accessed in voltage mode: *V*_OEND_ is swept from 0 V to 1.5 V and backward while *I*_OEND_ is measured. (b) Measured (symbols) and simulated (full line) electrical characteristic of the OEND accessed in current mode: *I*_OEND_ is swept and *V*_OEND_ is measured. The S‐shaped NDR region originates from the nonlinear current redistribution between the *R*_1_–*T*_1_–*R*_2_ and *R*_1_–*T*_2_ branches as *T*_2_ turns ON and *T*_1_ turns OFF. Dashed line is the load connected to the OEND. U denotes the operating point at the intersection of the *S*‐curve and the load line, which lies within the NDR region and drives self‐sustained oscillation. (c) Measured (symbols) and simulated (full line) spiking voltage *V*_spike_ over a 30 s window, confirming regular and stable oscillatory activity. The inset shows a 5 s detail of the waveform, resolving the shape of individual spikes. (d) Measured (blue line) and simulated (gray line) *V*_spike_ as a function of time, under a sinusoidal excitatory signal *V*_exc_(*t*) with 20 mV_pp_ amplitude and 30 s period. (e) Measured (blue line) and simulated (gray line) *V*_spike_ as a function of time under stepwise variation of the ion concentration *c*(*t*) at the load transistor *T*_3_. At *c* = 50 mM, the OAN remains quiescent. When *c* is reduced to 10 mM, the OAN spiking activity is restored. Returning to *c* = 50 mM reversibly suppresses oscillations, demonstrating full ionic reversibility.

To investigate the multimodality of the OAN, we modeled the electrical characteristic of the OECTs (Note S1) accounting for key material and device parameters: volumetric capacitance *C*_V_, ionic concentration‐dependent threshold voltage *V*_TH_, energy disorder γ, and channel length modulation λ. The model quantitatively reproduces the measured transfer and output characteristics of depletion‐mode and accumulation‐mode OECTs (Figures S3 and S4). Embedding this device model within a circuit simulator, we accurately captured both the OEND characteristics (Figure 2a,b) and the oscillatory spiking behavior of the OAN (Figure 2c). The close agreement between simulations and experiments not only validates the modeling framework but also provides a predictive tool for the rational design and systematic investigation of OAN architectures.

Returning to the current‐mode characteristic of Figure 2b, we now focus on the physical origin of the S‐NDR region that underlies the oscillatory behavior of the OAN. In current‐mode operation, *I*_OEND_ is the independent variable and *V*_OEND_ self‐adjusts according to the internal current partition between the two OEND branches. At low *I*_OEND_, *T*_1_ is ON and *T*_2_ is OFF; therefore, the imposed current mainly flows through the *R*_1_–*T*_1_–*R*_2_ branch. In this regime, *V*_OEND_ increases approximately linearly with *I*_OEND_, giving the low‐current positive‐resistance branch of the S‐shaped characteristic. As *I*_OEND_ increases, the voltage drop across *T*_1_ increases and, because of the OEND topology, *V*_SD1_ = *V*_SG2_. When *V*_SG2_ exceeds |*V*_TH2_|, *T*_2_ turns ON and the *R*_1_–*T*_2_ branch becomes conductive. Since *RT*_2_ is, by design, much smaller than *R*_2_, part of the imposed current is redirected from the *R*_1_–*T*_1_–*R*_2_ branch to the *R*_1_–*T*_2_ branch. This nonlinear current redistribution reduces the voltage required to sustain the imposed *I*_OEND_; therefore, *V*_OEND_ decreases while *I*_OEND_ continues to increase, generating the negative‐slope segment of the S‐NDR characteristic. With further increase of *I*_OEND_, the voltage drop across *R*_1_ increases because V*R*_1_ = *R*_1_ × *I*_OEND_. Since V*R*_1_ = *V*_GS1_, the overdrive of *T*_1_ is progressively reduced until *T*_1_ turns OFF. The current then flows predominantly through the *R*_1_–*T*_2_ branch, and *V*_OEND_ increases again with *I*_OEND_, restoring the high‐current positive‐resistance branch. Therefore, the current‐mode S‐NDR originates from the nonlinear partitioning of current between the *R*_1_–*T*_1_–*R*_2_ and *R*_1_–*T*_2_ branches and from the positive feedback mediated by *R*_1_, which couples the turn‐on of *T*_2_ to the turn‐off of *T*_1_. The three current‐mode regimes are summarized in Table S1.

The current‐mode S‐NDR characteristic is the relevant characteristic for the OAN dynamics because the active load and *C*_L_ probe the OEND through a load‐line condition. Sustained oscillation requires that the load line intersects the S‐NDR curve at a single unstable operating point lying within the negative‐slope region. To fulfill this condition, we employed a nonlinear active load formed by OECT *T*_3_ in series with resistor *R*_3_, whose characteristic is shown in Figure 2b (dashed line). The intersection between the OEND current‐mode characteristic and the active‐load line defines the operating point U. When U lies within the NDR region, the equilibrium is unstable, and the charging/discharging of *C*_L_ drives self‐sustained oscillation. This nonlinear active load offers a key advantage: its characteristic and hence the position of the operating point can be tuned on‐demand by adjusting the gate bias of *T*_3_. Specifically, the characteristic is governed by the resistance of the path *T*_3_, *R*_3_, and by the input voltage *V*_IN_ (Figure S5). At high *V*_OEND_, the voltage *V*_IN_–*V*_OEND_ is small, *T*_3_ operates in the linear region, and *R*_3_ is the dominant impedance element. As *V*_OEND_ decreases, the voltage *V*_IN_–*V*_OEND_ increases, *T*_3_ enters saturation, and the current becomes transistor‐limited. This crossover between resistor‐ and transistor‐limited regimes manifests as a characteristic change in the slope of the load curve and can be precisely controlled via *V*_G3_. We note that a purely resistive load would require a large resistance value to obtain a single intersection in the NDR region, which would increase the required operating voltage. Therefore, the series *T*_3_–*R*_3_ provides current limitation, voltage compatibility, and tunable load‐line positioning, thereby enabling direct control of the operating point U. When U falls within the NDR region, the OAN enters a self‐oscillatory regime, producing regular, continuous spiking over the entire 30 s measurement window, as shown in Figure 2c. The inset reveals the detailed waveform of individual spikes, confirming a spiking voltage *V*_spike_ of approximately 0.1 V with a stable oscillation amplitude.

The spiking activity of the OAN can be either triggered or suppressed by various inputs. One example is an excitatory signal *V*_exc_ superimposed on the input voltage *V*_IN_, as shown in Figure 2d. *V*_exc_ has a 20 mV peak‐to‐peak amplitude and a 30 s period. This signal modulates neuronal excitability by shifting the load line into or out of the NDR region. Upon excitation, phase‐locked bursting activity emerges synchronously with *V*_exc_, with the OAN returning to a quiescent state between consecutive bursts—faithfully reproducing the burst‐and‐silence alternation characteristic of biological neurons. The ionic concentration of *T*_3_ also modulates the OAN oscillatory dynamics, as shown in Figure 2e, where the NaCl concentration is varied within physiologically relevant conditions. The system remains in a stable quiescent state at 50 mM, whereas reducing the concentration to 10 mM drives the OAN into a bursting regime. Importantly, restoring the concentration to 50 mM reversibly suppresses the spiking activity, returning the OAN to its quiescent state, demonstrating the full reversibility of the ionic modulation. These results show that both electrical and ionic inputs can continuously tune the OAN between quiescent and bursting regimes, establishing the foundation for the multimodal mechano‐electrochemical response investigated in the following sections. Our model reproduces both the excitability and ion‐induced dynamics, as shown in Figure 2d,e (dark lines).

In the following, we examine how the OAN responds to applied strain and demonstrate how mechanical deformation can either excite or suppress its oscillatory activity. The effect of the mechanical stress is defined by the Cauchy strain formula *ε* = Δ*L*/*L*, where *ε* represents the mechanical strain, *L* is the object length, and Δ*L* is the elongation caused by the applied stress. To apply mechanical stress, we varied the radius of curvature (*r*), bending the OANs as depicted in Figure 3a, where red arrows indicate the direction of tensile strain experienced by *R*_1_, *R*_2_, and *R*_3_ upon downward bending. Since the thickness of the printed components is negligible compared to the substrate thickness (*t*_sub_ = 200 µm), the mechanical strain is given by [52, 53]:

(1) $$\varepsilon \left(\%\right)=100\frac{t_{\mathrm{sub}}}{2r}$$

**FIGURE 3:**
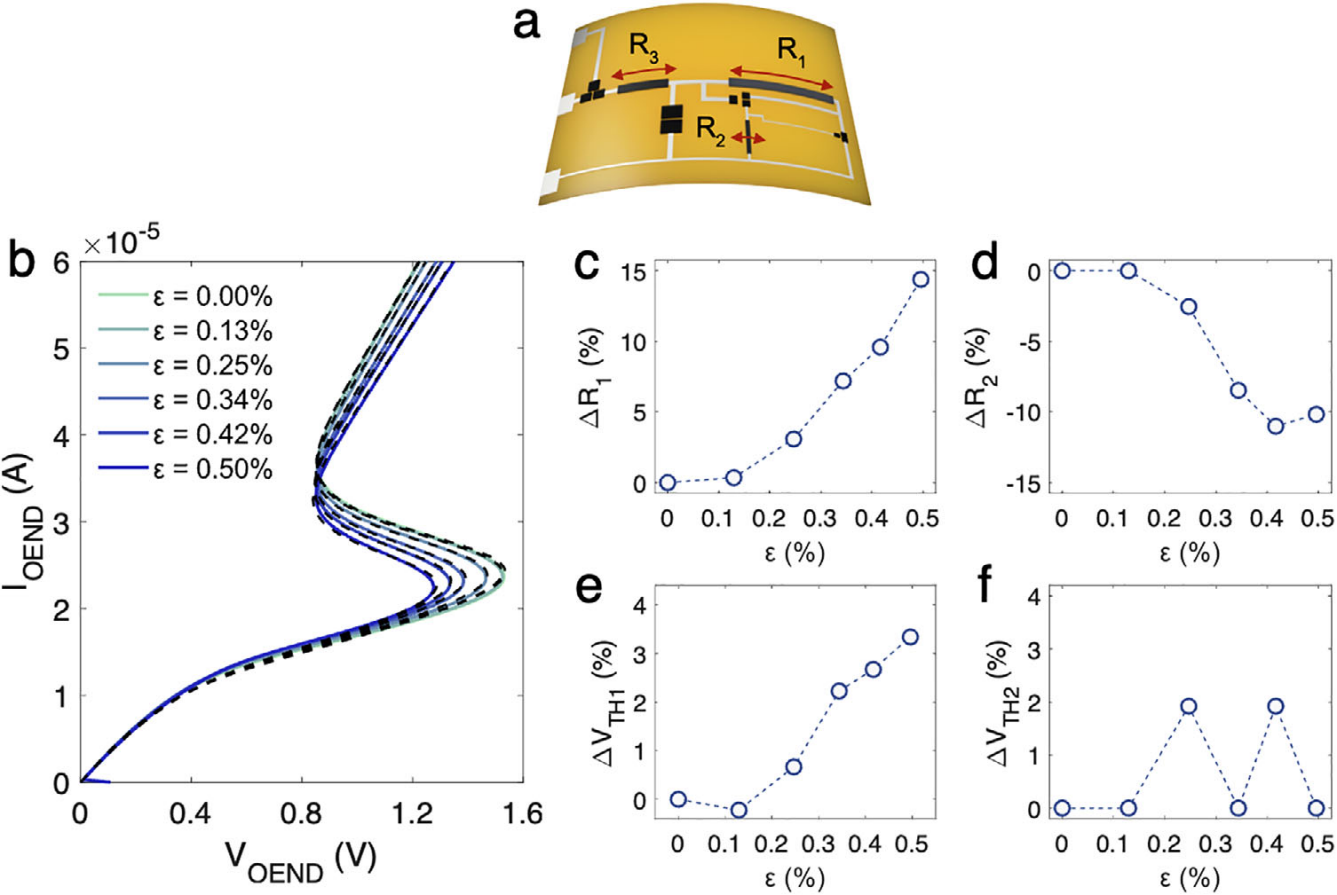
Strain‐dependent nonlinear electromechanical response of the OAN. (a) Schematic of the mechanical deformation applied to the OAN: downward bending induces tensile strain along *R*_1_ and *R*_3_ (aligned with the current flow direction, red arrows) and transverse strain across *R*_2_ (perpendicular to current flow), producing anisotropic resistance changes that reshape the OEND characteristic. (b) Measured (solid lines) and simulated (dashed lines) *I*_OEND_–*V*_OEND_ characteristics of the OEND in current mode for increasing mechanical strain *ε* = 0.00% to 0.50%, showing a progressive shift of the S‐NDR region. Percentage variation of (c) *R*_1_, (d) *R*_2_, (e) *V*_TH1_, and (f) *V*_TH2_ as a function of the mechanical strain *ε*. *R*_1_ increases monotonically by up to +15%, while *R*_2_ decreases by up to −13%, reflecting their opposite responses to tensile and transverse strain, respectively. *V*_TH1_ and *V*_TH2_ show comparatively minor changes compared to the resistor variations. The dominant mechanically induced modulation arises from strain‐dependent changes in the printed resistive elements, while OECT threshold‐voltage shift, *V*_TH1_, contributes to the local position of the S‐NDR switching boundaries and becomes relevant when the operating point U lies close to the NDR edge.

Figure 3b shows the progressive effect of mechanical strain on the OEND *S*‐curve where increasing strain (*ε* = 0.00% to 0.50%) systematically shifts the NDR region toward higher voltages. This strain‐induced reshaping of the *S*‐curve directly modulates the position of the operating point U relative to the NDR region, providing a mechanical handle on neuronal excitability. This behavior arises from strain‐induced changes in the electrical parameters of the device components, including deformation of the transistors and resistors.

To substantiate this interpretation, we incorporated mechanically induced parameter variations into our nonlinear electrochemical model and fitted the *S*‐curves measured under different strain conditions, achieving a quantitative description of the mechano‐electrical coupling. The simulation results (black dashed lines in Figure 3b) show excellent agreement with the experimental data. The isolated contribution of each parameter to the *S*‐curve and load line is systematically analyzed in Figure S6. This analysis shows that *R*_1_ predominantly controls the horizontal shift of the OEND S‐NDR curve because it sets the strength of the positive feedback acting on *T*_1_ (Figure S6a). *R*_2_ mainly governs the current amplitude of the high‐conductance branch, thereby affecting the current span of the S‐NDR window (Figure S6b). The threshold voltages *V*_TH1_ and *V*_TH2_ primarily affect the width and asymmetry of the NDR region (Figure S6d,e). Although the strain‐induced variation of *V*_TH1_ is comparatively small, *V*_TH1_ contributes to the switching boundaries of the OEND and therefore can slightly shift the edge of the NDR region, particularly when the operating point U is close to the transition between the negative‐slope and positive‐slope branches. In contrast, *R*_3_ does not modify the intrinsic OEND S‐NDR characteristic; rather, together with *T*_3_, it determines the slope and curvature of the active‐load line (Figure S6c) and therefore controls the position of U. This distinction is important because the OAN oscillatory state is determined by the relative position of U with respect to the NDR region.

The relative variations of the main OAN parameters as a function of the mechanical strain are displayed in Figure 3c–f, revealing that *R*_1_ increases monotonically by up to +15% at *ε* = 0.50%, while *R*_2_ decreases by up to −13%, and the threshold voltages *V*_TH1_ and *V*_TH2_ exhibit comparatively minor variations of less than 4%. Their absolute values are shown in Figure S7, where *R*_1_ spans from ∼14.5 to ∼17 kΩ and *R*_2_ decreases from ∼120 to ∼105 kΩ across the measured strain range, while *V*_TH1_ increases monotonically from ∼449 to ∼467 mV and *V*_TH2_ remains approximately constant at ∼52 mV.

These parameter dependences provide practical design rules for selecting the passive elements and bias conditions of the OAN. *R*_1_ and *R*_2_ are first selected to generate an OEND S‐NDR characteristic with a sufficiently broad voltage and current window, while *R*_3_, *T*_3_, *V*_G3_, and *V*_IN_ position the active‐load line relative to this window. Oscillations occur when the operating point U lies inside the NDR region, whereas silent operation is obtained when U lies outside it. Thus, the design target is a parameter space, rather than a single finely tuned parameter set. To quantify this region, we introduced a normalized two‐dimensional NDR margin, M_B,2D_ (Note S2), defined as the signed distance between U and the closest boundary point of the NDR segment in the *V*_OEND_–*I*_OEND_ plane. The resulting parameter maps show an extended oscillatory region around the nominal *R*_1_–*R*_2_ design and confirm that *V*_G3_ provides an effective tuning parameter to move the active‐load line across the NDR boundary (Figure S8). Monte Carlo simulations further show that the spiking yield remains above 89% for all single‐parameter variations except *V*_TH3_, which is the most critical parameter as it controls the load‐line. This sensitivity can be compensated through *V*_G3_, restoring the target spiking condition and substantially improving the all‐parameter spiking yield from 26.47% to 67.90% (Figure S9).

Downward bending induces tensile strain in all resistors within the elastic regime. As shown in Figure 3c–f, the dominant strain‐dependent variations occur in *R*_1_ and *R*_2_. More specifically, *R*_1_ shows a pronounced and monotonic increase in resistance because the tensile stress is aligned with the current flow, while *R*_2_ shows an opposite trend as the applied strain is perpendicular to the transport direction. Together, these results establish a clear physical picture: mechanical deformation selectively and anisotropically modifies *R*_1_ and *R*_2_, which in turn shift the S‐NDR characteristic and thereby control the excitability threshold of the OAN, linking substrate strain directly to neuronal dynamics. Therefore, the OAN should be understood as a mechanically tunable nonlinear organic electrochemical circuit with neuron‐like excitability, rather than as a direct material‐level analogue of a mechanosensitive ion channel.

Figure 4a shows an OAN operating in a mechanically induced inhibition regime. In the absence of bending, the device exhibits sustained spiking activity, which is progressively suppressed upon substrate deformation. Stepwise increases in mechanical strain are applied at the times marked by arrows, with resting phases highlighted in green; between consecutive strain steps, the OAN is allowed to relax before the next deformation is applied. The applied stress induces strain that builds up asymptotically toward its maximum value, introducing a delay between mechanical actuation and the corresponding change in oscillatory behavior. The OAN continues to spike until the strain reaches *ε* ≈ 0.42%; above this threshold, oscillations are suppressed. A strain of 0.5%, applied approximately 60 s after the beginning of the experiment and maintained for 10 s, completely silences the neuron. Because strain relaxation is not instantaneous, the recovery of bursting activity in the final resting phase is correspondingly delayed. This temporal memory of the mechanical state mirrors the adaptation kinetics of biological mechanoreceptors and may be exploited in future designs to encode stimulus duration or rate‐of‐change into the neuronal firing pattern. The model reproduces this dynamic behavior by explicitly accounting for the time‐dependent development and release of strain. Figure S10 shows that *f*_spike_ increases monotonically from 0% to 67% of its maximum value as *ε* increases from 0.13% to 0.50% within the spiking regime.

**FIGURE 4:**
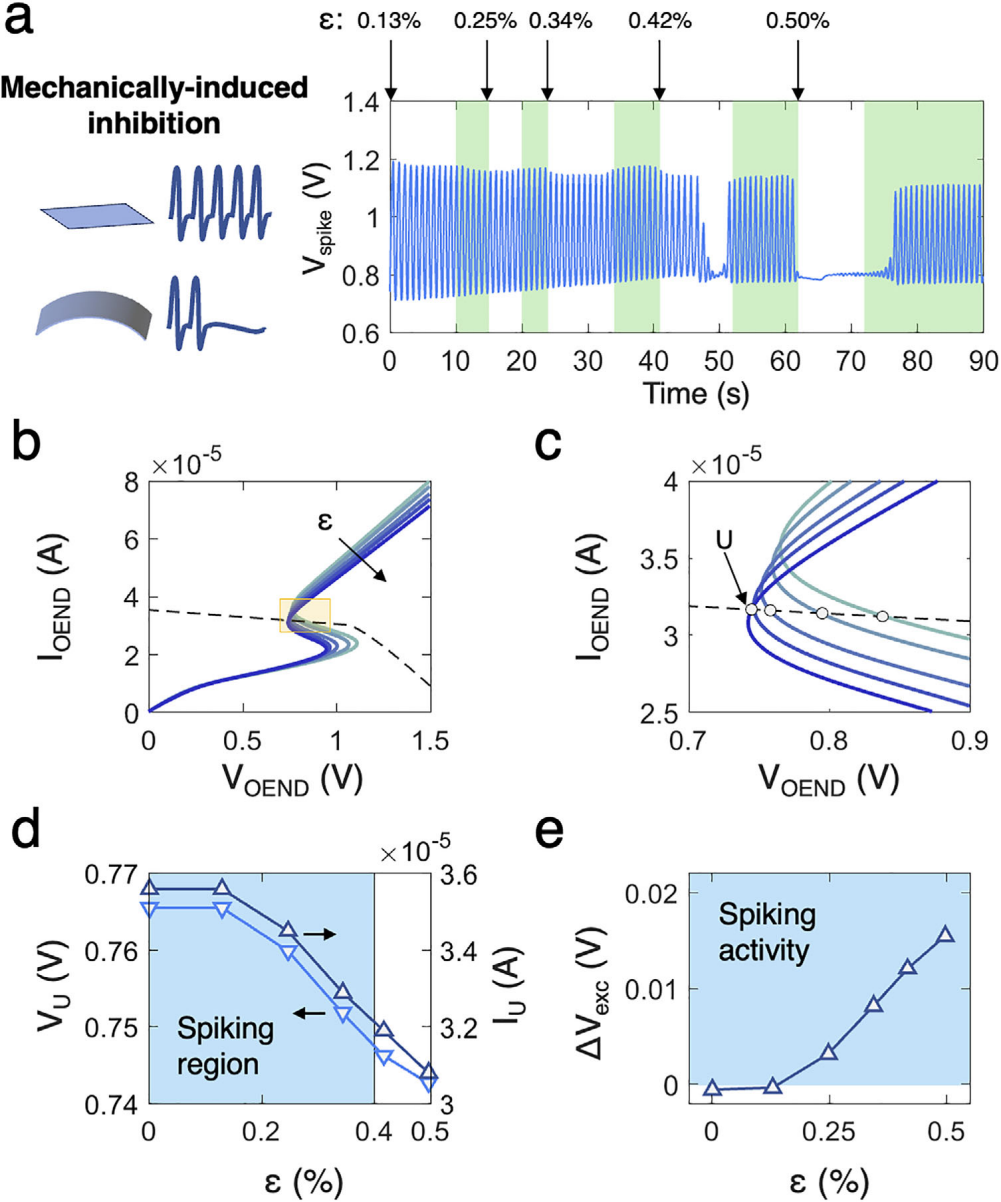
Mechanically induced inhibition. (a) Measured *V*_spike_ as a function of time under stepwise increasing mechanical strain. Green regions indicate resting phases with no applied bending; arrows mark the onset of each deformation step. The OAN oscillates at low strain and is silenced above *ε* ≈ 0.42%. (b) *S*‐curves of the OEND for increasing *ε* (solid lines, light green to dark blue) and the active load line (black dashed). The load line is positioned near the upper boundary of the NDR region, so that sufficiently large strain shifts U outside the spiking region. (c) Zoom of the operating point (yellow box in panel b): when U intersects the *S*‐curve on its negative‐slope branch (green curves), the OAN spikes. When U moves to the positive‐slope branch (blue curves), oscillations are suppressed. (d) Operating point voltage *V*_U_ (left axis) and current *I*_U_ (right axis) as a function of *ε*. Within the light blue spiking region, both *V*_U_ and *I*_U_ decrease monotonically; above the threshold strain, U exits the NDR region, and the OAN is silenced. (e) Excitatory voltage Δ*V*_exc_ as a function of *ε*. Negative values indicate that U is already within the spiking region while positive values quantify the electrical signal needed to re‐excite the neuron after mechanically induced inhibition.

The relative parameter variations extracted from the strain‐dependent fits (Figure 3c–f) were implemented in the numerical model to reproduce the strain‐dependent behavior. The simulated time response presented in Figure S11a closely matches the experimental data of Figure 4a, enabling a mechanistic interpretation of the strain‐induced modulation. Figure 4b presents the simulated *S*‐curves under different strain levels, directly linking mechanical deformation to the nonlinear electrochemical response. The load line (black dashed curve) intersects the *S*‐curve at an operating point U, whose position depends on *ε*, as detailed in Figure 4c. As strain increases, U shifts from within the NDR region to the upper positive‐slope branch of the *S*‐curve. When *ε* exceeds ≈0.42%, the operating point enters this stable region, suppressing oscillations. This transition is summarized in Figure 4d, where the voltage and current at U are plotted as a function of *ε*. Both *V*_U_ and *I*_U_ decrease monotonically with increasing *ε*, with the boundary of the spiking regime (light blue area) corresponding to the strain at which U reaches the upper edge of the NDR region. For *ε* below this threshold, U lies within the NDR region and the OAN oscillates; above it, the operating point shifts to the stable branch and spiking is inhibited.

The position of the operating point U, and hence the spiking threshold, can be precisely and continuously controlled by the gate voltage *V*_G3_ of OECT *T*_3_. Figure S12 illustrates how *V*_G3_ modulates the load line, shifting it vertically and thereby repositioning U along the *S*‐curve. Since *T*_3_ is a depletion‐mode transistor, increasing *V*_G3_ reduces the source‐gate voltage and thereby lowers the drain current, pulling the load curve downward. This provides a direct electrical handle over whether U falls within or outside the NDR region. We define the excitatory voltage Δ*V*_exc_ as the increment in *V*_G3_ required to position U at the upper edge of the NDR region, that is, the transition point where the *S*‐curve slope changes from negative to positive. This quantity is particularly useful for determining the threshold to re‐excite the neuron after its bursting activity has been inhibited by mechanical strain. As shown in Figure 4e, Δ*V*_exc_ is negative for *ε* < 0.42% (U already within the NDR region), approximately zero at *ε* ≈ 0.42% (U at the upper transition point), and increases monotonically for *ε* > 0.42%, quantifying the electrical stimulus required to overcome the mechanically induced inhibition.

By appropriately configuring the load line through *V*_G3_, the OAN can be programmed to operate in a mechanically induced excitation regime. In this configuration, the device is silent at rest and becomes active only upon substrate deformation, as mechanical strain shifts U into the NDR region and triggers oscillations. This strain‐induced activation demonstrates that neuronal excitability can be directly encoded in the device physics, enabling mechanical stimuli to act as the primary driver of spiking activity [41, 42, 43]. This complementary regime is experimentally demonstrated in Figure 5. Figure 5a shows that the OAN remains silent in the absence of strain and transitions to sustained bursting activity under stepwise increasing mechanical deformation, with quiescent intervals highlighted in green. Notably, the onset of spiking occurs at *ε* ≈ 0.25% and persists through *ε* = 0.50%, in contrast to the inhibition case where spiking is suppressed above *ε* ≈ 0.42%, thus reflecting the different load line configurations in the two regimes. Figure S10 shows that *f*_spike_ increases monotonically from 0% to 67% of its maximum value as *ε* increases from 0.13% to 0.50% within the spiking regime. Simulations based on the strain‐dependent parameters closely reproduce the observed dynamics (Figure S11b).

**FIGURE 5:**
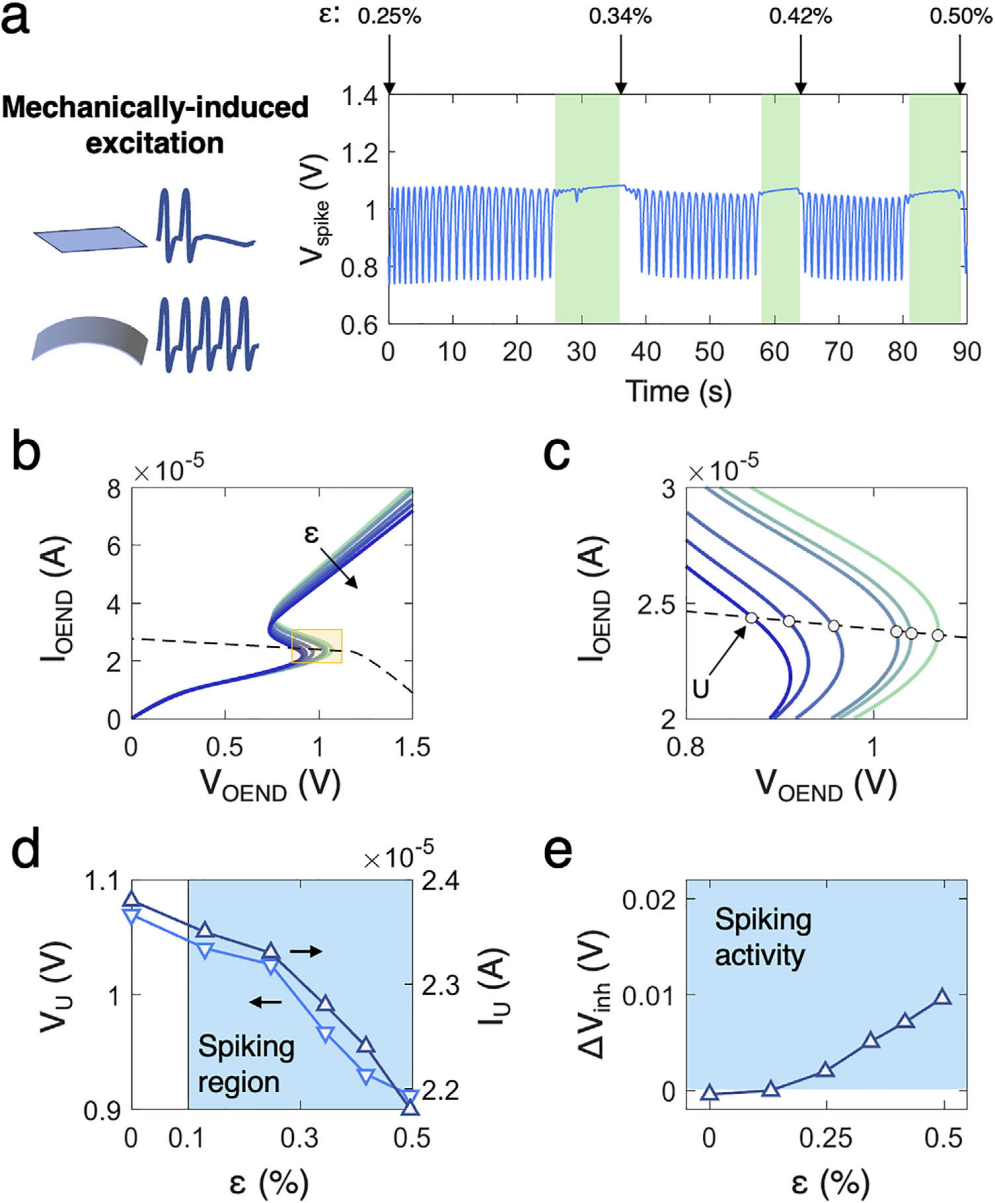
Mechanically induced excitation. (a) Measured *V*_spike_ as a function of time under stepwise increasing mechanical strain. Green regions indicate resting phases with no applied bending; arrows mark the onset of each deformation step. The OAN is silent at rest and transitions to sustained bursting above *ε* ≈ 0.25%. (b) *S*‐curves of the OEND for increasing *ε* (solid lines, light green to dark blue) and the nonlinear load line (black dashed). The load line is positioned near the lower boundary of the NDR region, so that sufficiently large strain shifts U from a stable branch into the spiking region. (c) Zoom of the operating point (yellow box in panel b): when U intersects the *S*‐curve on its negative‐slope branch (blue curves), the OAN spikes. When U lies on the positive‐slope branch (green curves), the device remains silent. (d) Operating point voltage *V*_U_ (left axis) and current *I*_U_ (right axis) as a function of *ε*. Below the threshold strain, U lies outside the NDR region and the OAN is silent. Above it, U enters the light blue spiking region and oscillations are triggered. Both *V*_U_ and *I*_U_ decrease monotonically with increasing *ε* within the spiking regime. (e) Inhibitory voltage Δ*V*_inh_ as a function of *ε*. Δ*V*_inh_ increases monotonically with *ε*, indicating that larger deformation drives U deeper into the NDR region and requires stronger electrical counteraction to restore the silent state.

The underlying mechanism is illustrated in Figure 5b–e. In this configuration, the load line is positioned at the lower edge of the NDR region. As strain increases, the *S*‐curve shifts rightward and the operating point U moves progressively from a stable branch at low *ε* into the NDR region (Figure 5c), thereby triggering spiking activity. The zoom in Figure 5c confirms that U transitions from outside to inside the NDR region as *ε* increases from 0.25% to 0.50%, with each successive operating point displaced further into the unstable region. Consistently, Figure 5d shows that both *V*_U_ and *I*_U_ evolve with *ε* such that the system transitions from outside to inside the spiking region. The spiking threshold can be quantitatively tuned through *V*_G3_. We define the inhibitory voltage Δ*V*_inh_ as the increase in gate voltage required to move U to the lower boundary of the NDR region and suppress strain‐induced oscillations. As shown in Figure 5e, Δ*V*_inh_ increases monotonically with *ε* within the spiking regime, indicating that larger mechanical deformation drives U deeper into the NDR region and therefore requires a stronger electrochemical stimulus to restore the silent state.

The ability to obtain both mechanically induced inhibition and excitation from the same OAN highlights that bending acts by repositioning the operating point U relative to the S‐NDR region, rather than by imposing a fixed neuronal response. This response is not isotropic and depends on how the printed resistive elements are oriented with respect to the strain field. To examine this layout dependence, we investigated an orthogonal‐bending configuration, in which the strain field is rotated by 90° with respect to the original device orientation. The results are summarized in Figure S13.

In this orthogonal configuration, the longitudinal and transverse strain contributions acting on *R*_1_ and *R*_2_ are interchanged. Simulations based on the same nonlinear OAN model show that this redistribution reverses the direction of the S‐NDR displacement. Thus, orthogonal bending moves the S‐NDR characteristic in a complementary direction with respect to the original bending configuration. The neuronal response is then set by the relative position of the active‐load line. When the load line is placed so that increasing strain drives U out of the NDR region, orthogonal bending suppresses the oscillations (Figure S13b,c). Conversely, when the load line is configured so that increasing strain drives U into the NDR region, the same orthogonal bending stimulus triggers spiking (Figure S13f,g).

The *V*_spike_‐time traces confirm both regimes. In the inhibition configuration (Figure S13d), stepwise orthogonal bending progressively suppresses the oscillatory activity. In the excitation configuration (Figure S13h), the OAN is silent at rest and starts spiking under orthogonal bending. These results show that orthogonal bending does not eliminate the mechanical control of excitability. Instead, it provides a complementary layout configuration in which the direction of the S‐NDR displacement is reversed, while inhibition or excitation can still be programmed by repositioning the active‐load line through the bias conditions.

Overall, these results demonstrate that the mechanosensitivity of the OAN is programmable and layout‐dependent. Mechanical stimuli can be deterministically converted into either excitatory or inhibitory neuronal responses by positioning the operating point U relative to the S‐NDR region through the active‐load configuration. Moreover, the orthogonal‐bending analysis shows that the direction of the strain‐induced S‐NDR displacement can be engineered through the relative orientation between the bending direction and the printed resistive elements. This layout‐level programmability provides an additional design degree of freedom for mechanically tunable OANs.

The proposed architecture defines a general design framework for mechanically and chemically sensitive OANs. Excitability is governed by the relative displacement between the OEND S‐NDR characteristic and the active‐load line, which sets the position of the operating point U. Scaling this framework does not require preserving the absolute dimensions of the printed layout, but rather the normalized strain‐induced parameter shifts and the load‐line positioning that move U across the NDR boundary. Smaller footprints can first be pursued by placing the OECTs, resistors, capacitor, and interconnects closer together while preserving the same circuit topology. The scaling of the strain‐sensitive resistive elements can be rationalized from first‐order geometrical considerations. For a printed resistor strained along its length, *ε* = Δ*L*/*L* and *R* = *ρ* × *L*/(*W* × *t*), where *ρ*, *L*, *W*, and *t* are the resistivity, length, width, and thickness of the printed conductor, respectively. Therefore, Δ*R* = *R* × *ε*. This relation shows that, for a given strain, the resistance modulation can be preserved if the resistance *R* is preserved during scaling. This can be achieved by maintaining the relevant geometrical ratios *L*/(*W* × *t*), or by using higher‐resistivity materials, thinner films, or larger sheet resistance. Compact, oriented, or serpentine resistor geometries can further preserve the desired strain sensitivity and directionality in smaller footprints. Analogous considerations hold for OECTs, and the S‐NDR/load‐line framework provides practical scaling guidelines for compact mechano‐chemical OANs, as summarized in Table S2.

Building on this programmable mechanosensitivity, we demonstrate multimodal operation of the OAN by combining mechanical strain *ε* and electrolyte concentration c as independent and simultaneous control parameters. To clarify their respective roles, we distinguish between the intrinsic OEND S‐NDR characteristic and the active‐load characteristic. The OEND S‐NDR curve is determined by *T*_1_, *T*_2_, *R*_1_, *R*_2_, and their threshold voltages: *R*_1_ mainly controls the positive feedback acting on *T*_1_ and shifts the S‐NDR curve, *R*_2_ sets the current level of the high‐conductance branch, while *V*_TH1_ and *V*_TH2_ define the switching boundaries and asymmetry of the NDR region. In contrast, the active‐load line is determined by *T*_3_, *R*_3_, *V*_G3_, *V*_IN_, and the ion concentration in the electrolyte of *T*_3_. *R*_3_ sets the load‐line slope, whereas *T*_3_ provides nonlinear current limitation. The electrolyte concentration of OECT *T*_3_ modulates its threshold voltage *V*_TH3_ through the concentration‐dependent electrolyte potential described in Note S1 and Equations (S7–S9), which includes Nernst and Donnan contributions. As a result, changing the ion concentration c modifies the effective gate overdrive *V*_G3_−*V*_TH3_(*c*), thereby altering the drain current of *T*_3_ and shifting the active‐load line. Mechanical strain, instead, reshapes the OEND S‐NDR curve through strain‐dependent variations of the OEND parameters. Thus, the two stimuli act on distinct circuit characteristics: mechanical strain displaces the S‐NDR curve, whereas electrochemical modulation of *T*_3_ displaces the load line. Their interplay determines the position of the operating point U relative to the NDR region and, consequently, the neuronal state, enabling programmable control over excitation and inhibition. A component‐level summary of these effects is provided in Table S3.

We first consider the configuration in which mechanical stimulation induces excitation and chemical input suppresses it (Figure 6a–e). More specifically, Figure 6d shows that the neuron is initially silent at low *ε* and low c because U lies on the stable positive‐slope branch (Figure 6e, left panel). When mechanical strain (*ε* = 0.35%) is applied (Figure 6b), the *S*‐curve shifts and U moves into the NDR region, triggering oscillations (Figure 6d, middle window; Figure 6e, middle panel). Increasing the ion concentration from 100 to 500 mM (Figure 6c) shifts the load line downward, moving U back to the stable branch and inhibiting spiking (Figure 6d, right window; Figure 6e, right panel). Video S1 provides a real‐time demonstration of this sequence. The three *I*–*V* panels of Figure 6e thus trace a complete silent‐to‐spiking‐to‐silent cycle driven by mechano‐chemical stimuli.

**FIGURE 6:**
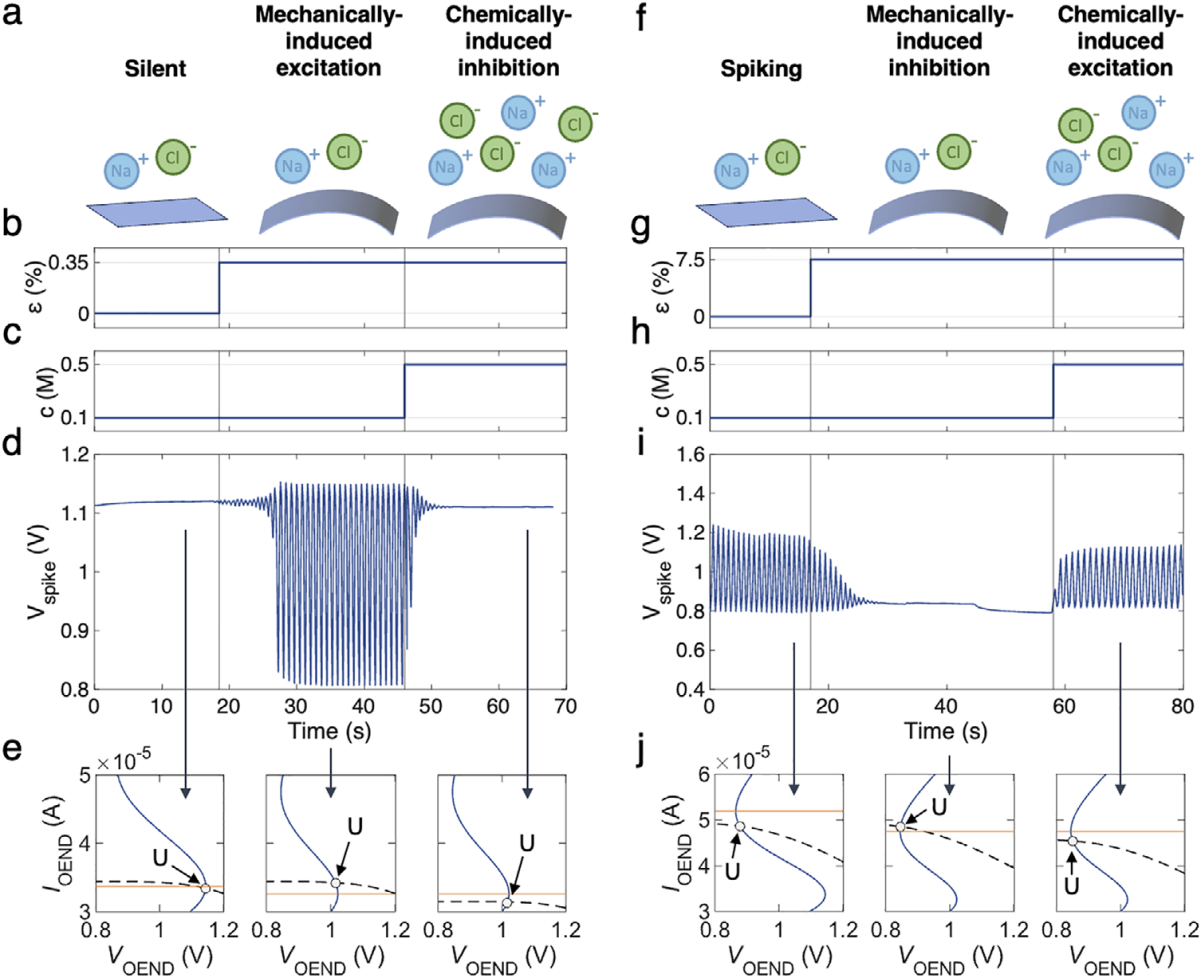
Multimodal mechano‐electrochemical control of OAN activity. (a–e) Mechanically induced excitation followed by chemically induced inhibition. (a) Schematic of the operating regime: the OAN is silent at rest, mechanical strain triggers bursting, and subsequent increase of ionic concentration suppresses it. (b) Applied mechanical strain *ε* as a function of time: a step to *ε* = 0.35% is maintained throughout the second and third phases. (c) Impact of the ion concentration: after mechanical excitation is established, c is increased from 100 to 500 mM to chemically inhibit the OAN. (d) Measured *V*_spike_ as a function of time, showing the full silent‐to‐spiking‐to‐silent sequence driven by mechanical and chemical stimuli. (e) *I*_OEND_–*V*_OEND_ characteristics (solid line) and load line (dashed line) for each of the three states in panel d. In the middle panel, mechanical strain shifts the OEND S‐NDR characteristic with respect to the unchanged load line, moving U into the NDR region. In the right panel, the increased ion concentration at *T*_3_ modifies *V*_TH3_ and the effective gate overdrive *V*_G3_−*V*_TH3_(*c*), thereby shifting the active‐load line and moving U back to the stable branch. The orange horizontal line marks the upper boundary of the NDR region. (f–j) Mechanically induced inhibition followed by chemically induced excitation. (f) Schematic of the complementary operating regime: the OAN oscillates at rest, mechanical strain suppresses bursting, and subsequent increase of ionic concentration restores firing activity. (g) Applied mechanical strain *ε* as a function of time: a step to *ε* = 0.35% is maintained throughout the second and third phases. (h) Impact of the ion concentration: after mechanical inhibition is established, c is increased from 100 to 500 mM to chemically re‐excite the OAN. (i) Measured *V*_spike_ as a function of time, showing the complementary spiking‐to‐silent‐to‐spiking sequence. (j) *I*_OEND_–*V*_OEND_ characteristics (solid line) and load line (dashed line) for each of the three states in panel i. In the middle panel, mechanical strain shifts the OEND S‐NDR characteristic with respect to the unchanged load line, moving U out of the NDR region. In the right panel, the increased ion concentration at *T*_3_ modifies *V*_TH3_ and *V*_G3_−*V*_TH3_(*c*), thereby shifting the active‐load line and restoring U inside the NDR region. The orange horizontal line marks the upper boundary of the NDR region. A truth‐table summary of the corresponding multimodal spiking/silent states is reported in Table S4.

We then demonstrate the complementary regime (Figure 6f–j), in which mechanical strain inhibits spiking and chemical input restores it. Figure 6i shows that the OAN initially oscillates at low *ε* and c because U lies within the NDR region (Figure 6j, left panel). Applying mechanical strain shifts the OEND S‐NDR curve with respect to the unchanged load line, so that U exits the NDR region and bursting is suppressed (Figure 6i, middle window; Figure 6j, middle panel). The subsequent increase of ion concentration c modifies *V*_TH3_ and the effective gate overdrive *V*_G3_−*V*_TH3_(*c*) of *T*_3_, shifts the active‐load line, and drives U back into the NDR region, restoring spiking activity (Figure 6i, right window; Figure 6j, right panel). The *I*–*V* sequence in Figure 6j thus traces the complementary spiking‐to‐silent‐to‐spiking cycle, confirming the full reversibility and programmability of the multimodal response. The corresponding spiking/silent states for all input combinations are summarized in Table S4, which provides a truth‐table representation of the multimodal OAN response.

Together, these results establish that mechanical and chemical stimuli act as synergistic and independently programmable control parameters of neuronal excitability. The same physical device can be reconfigured to produce opposite neuronal responses to the same stimulus combination, demonstrating true multimodal neuromorphic functionality at the single‐device level.

## 3 Conclusions

In this work, we have demonstrated OANs monolithically integrated on flexible substrates and established nonlinear mechano‐electrochemical coupling as the physical mechanism underpinning multimodal neural operation. Depending on the operating point configuration, mechanical deformation and electrochemical stimuli—applied independently or concurrently—deterministically drive the OAN between quiescent and spiking regimes through controlled shifts of the nonlinear characteristic across the current–voltage plane. This programmable multimodal behavior enables excitation or inhibition to be encoded directly in the device physics, with ionic concentration and mechanical strain serving as tunable control parameters. Quantitative agreement between experiments and numerical simulations of the governing mechano‐electrochemical nonlinear dynamics validates a predictive modeling framework for this device class and provides a mechanistic basis for the rational design of OAN architectures.

The results reported here advance the field along three interconnected axes. At the device level, we establish that the S‐NDR characteristic of an OECT‐based nonlinear circuit can be reshaped on‐demand by substrate deformation, providing a direct link between mechanical stimuli and neuronal dynamics that was previously missing. This mechanosensitivity is functional rather than molecular: mechanical deformation controls neuron‐like excitability by tuning a nonlinear organic electrochemical circuit. At the circuit level, we demonstrate that the position of the neuronal state can be precisely programmed through the interplay of mechanical strain, ionic concentration, and gate bias, enabling four‐state programmable control over excitation and inhibition from a single OAN device. Importantly, the direction and magnitude of the strain‐induced S‐NDR displacement can be engineered through the relative orientation between the strain field and the printed OAN components, providing a layout‐level design parameter for programming mechanically induced excitation and inhibition. At the systems level, we show that mechanical and electrochemical inputs act as synergistic and independently programmable control variables, capturing selected functional features of biological mechanosensitive neurons in a fully organic, flexible platform.

Collectively, these advances establish a rigorous device‐ and circuit‐level basis for multimodal OANs capable of programmable mechano‐electrochemical control of excitability. The present work demonstrates this principle in a two‐input mechano‐electrochemical OAN, while future implementations may extend the S‐NDR/load‐line framework to additional layouts, sensing elements, and input modalities. Such extensions will require dedicated device and circuit co‐design, but the results presented here define the operating principles for this development. Beyond serving as a neuromorphic building block, the OAN introduced here represents a physically grounded model system for studying mechano‐electrochemical nonlinear dynamics in soft matter, which is an area with broad relevance to bioelectronics, soft robotics, and physiological sensing. Looking forward, these findings open concrete pathways toward embodied intelligence in event‐based sensorimotor systems, with direct implications for bio‐inspired robotics, wearable health monitoring, and implantable neuromorphic interfaces.

## 4 Experimental Section

### 4.1 Materials

PEDOT:PSS (Clevios PH500) and its co‐reagents (3‐glycidyloxypropyl) trimethoxysilane (GOPS), ethylene glycol (EG), sorbitol, and diethylenetriamine (DETA) were all procured from Sigma‐Aldrich. The following materials were also procured from Sigma‐Aldrich: thin polyimide (PI) Kapton single‐sided tape, poly(sodium‐4‐styrene sulfonate) (PSS:Na) powder (*M*_w_ = 1 000 000), 4‐dodecylbenzenesulfonic acid (DBSA), and NaCl salt.

Polymer and polyelectrolyte formulations: PEDOT:PSS was filtered (0.22 µm filter) and readily mixed with 5% (v/v) EG, 1% (v/v) GOPS and 4.6 mg mL^−1^ sorbitol to achieve the depletion‐mode formulation (PEDOT‐D). The ink was vigorously mixed for 10 min after the addition of the co‐reagents, and each time before filling the printing syringe.

PEDOT:PSS accumulation‐mode (PEDOT‐A) was obtained from the previous formulation following the addition of 33% (v/v) DETA, molecule used as an amino‐based de‐doping reagent [35]. After the incorporation of DETA, the ink was subjected to a second round of mixing for 10 min and then placed in an ultrasonic bath for 5 min.

Polyelectrolyte was prepared from 24 mg of PSS:Na powder in 1 mL of deionized (DI) water. 1% (v/v) HCl (1 M), 2.5% (v/v) GOPS and 0.25% (v/v) DBSA were added to the mixture, which was mixed for 30 min before use.

### 4.2 Fabrication of the Building‐Block OAN Components

PI was first put on glass slides and rinsed with acetone, DI water, and isopropanol. The substrate was then dried with nitrogen gas and treated in an ultraviolet UV‐ozone chamber for 5 min. All the components (OECTs, resistors, and capacitors) were printed on the PI using additive manufacturing via ultra‐precise micro‐dispensing. Several parameters define the printing resolution, namely the printing speed, the traveling (noncontact) speed, and the needle size. These parameters differ for each ink type and are reported in Table S5. OECT source and drain electrodes were patterned from conductive carbon paste. PEDOT‐D or PEDOT‐A was then deposited between the two electrodes to build the conductive channels of the depletion and accumulation mode devices, respectively. A third electrode of Ag/AgCl was printed near the channel to form the coplanar gate. The resistor lines were printed with carbon paste. The capacitor was fabricated with carbon pads covered by PEDOT‐D. The two pads were ionically connected with a PSS:Na layer hydrated with a 1 M NaCl solution. The interconnections and connecting pads were made with silver paste and covered with encapsulant paste to prevent oxidation and conductivity loss. Silicone wells were patterned directly on the substrate to contain the electrolyte. They were left to dry at room temperature for 20 min before the measurements.

### 4.3 Electrical Measurements

OEND and OECT characteristics were measured with a Keithley 2602B dual source meter unit. The gate‐to‐source voltage was linearly swept between −0.4 and 0.4 V at a fixed drain‐to‐source voltage of 0.1 V drain‐to‐source voltage for the transfer characteristics. The output curves were obtained sweeping the drain‐to‐source voltage from −0.4 to 0 V at fixed gate‐to‐source voltages (−0.4, −0.2, 0, 0.2, and 0.4 V). Unless otherwise specified, measurements were performed in 0.1 M NaCl. OAN DC and alternating current (AC) operations were finally measured with a Digilent Analog Discovery 3 oscilloscope and waveform generator. Electrochemical impedance spectroscopy (EIS) characterizations were performed in two‐electrode setup to extract the capacitance of the printed capacitor using a PalmSens4 potentiostat.

### 4.4 Numerical Simulations

The OECT model described in Note S1 was integrated into a Verilog‐A module for use in OAN simulations with Advanced Design System (ADS) 2025 Update 1, Keysight Technologies. To address the mechanical strain (*ε*) exerted on the OAN, parametric simulations were performed on the relevant parameters. Design‐window maps were obtained by sweeping *R*_1_, *R*_2_, *R*_3_, and *V*_G3_, and evaluating the normalized 2D NDR margin M_B,2D_. Monte Carlo tolerance simulations were performed by sampling the relevant circuit parameters from Gaussian distributions centered at their nominal values with standard deviation equal to 10% of the nominal value. For single‐parameter Monte Carlo simulations, 5000 samples were used for each parameter; for all‐parameter Monte Carlo simulations, 10 000 samples were used. Data analysis was conducted using MATLAB 2024a, MathWorks.

## Author Contributions

**Rassen Boukraa**: investigation, validation, writing – review and editing, visualization, formal analysis, data curation. **Pietro Belleri**: investigation, writing – original draft, visualization, validation, formal analysis, data curation. **Zsolt M. Kovács‐Vajna**: writing – original draft, supervision, visualization, formal analysis. **Paschalis Gkoupidenis**: writing – original draft, formal analysis, validation, visualization, methodology. **Fabrizio Torricelli**: writing – original draft, writing – review and editing, conceptualization, funding acquisition, validation, visualization, formal analysis, project administration, supervision, resources, methodology.

## Funding

The authors R.B., P.B., Zs.M.K‐V, and F.T. acknowledge the financial support of the project “Smarcap”, funded by Ministero dello Sviluppo Economico (MISE), now Ministero delle Imprese e del Made in Italy (MIMIT), Fondo per lo sviluppo di tecnologie e applicazioni di intelligenza artificiale, blockchain e internet of things, (FTE0000034), C.U.P. B87H21012300008. The authors R.B., P.B., and F.T. acknowledge the financial support of the project “Tecnologie portatili e protocolli innovativi per la diagnosi ultrasensibile di Xylella fastidiosa direttamente in piante e vettori” (1LIVEXYLELLA), C.U.P. H93C22000760006, funded by Ministero dell'agricoltura, della sovranità alimentare e delle foreste. The authors P.B., and F.T. acknowledge the financial support of the project “Research actions for reducing the impact on agricultural and natural ecosystems of the harmful plant pathogen Xylella fastidiosa” (REACH‐XY), C.U.P. B93C22001920001, funded by legge di bilancio del 30 dicembre 2021, n. 234, Consiglio Nazionale delle Ricerche. The author P.G. acknowledges funding from the Carl‐Zeiss‐Stiftung, via the Emergent AI Center of Johannes Gutenberg University, Mainz, Germany.

## Conflicts of Interest

The authors declare no conflicts of interest.

## Supporting information




**Supporting File 1**: adma75027‐sup‐0001‐SuppMat.pdf.

**Supporting File 2**: adma75027‐sup‐0002‐VideoS1.mp4.

## Data Availability

The data that support the findings of this study are available from the corresponding authors on request.
