# Supplementary material for: Bioinspired Mechanosensitive Organic Artificial Neuron With Programmable Excitatory and Inhibitory Responses via Nonlinear Mechano‐Electrochemical Coupling
Source: Adv Mater. 2026 Sep 17;38(61):e75027. doi: 10.1002/adma.75027 (PMC13726698; doi:10.1002/adma.75027)
Supplement: Supplementary file 1 [file ADMA-38-e75027-s002.pdf]

Supporting Information

Bioinspired Mechanosensitive Organic Artificial Neuron with Programmable Excitatory and Inhibitory Responses via Nonlinear Mechano-Electrochemical Coupling

*Rassen Boukraa, Pietro Belleri, Zsolt M. Kovács-Vajna, Paschalis Gkoupidenis, Fabrizio Torricelli**

E-mail: fabrizio.torricelli@unibs.it

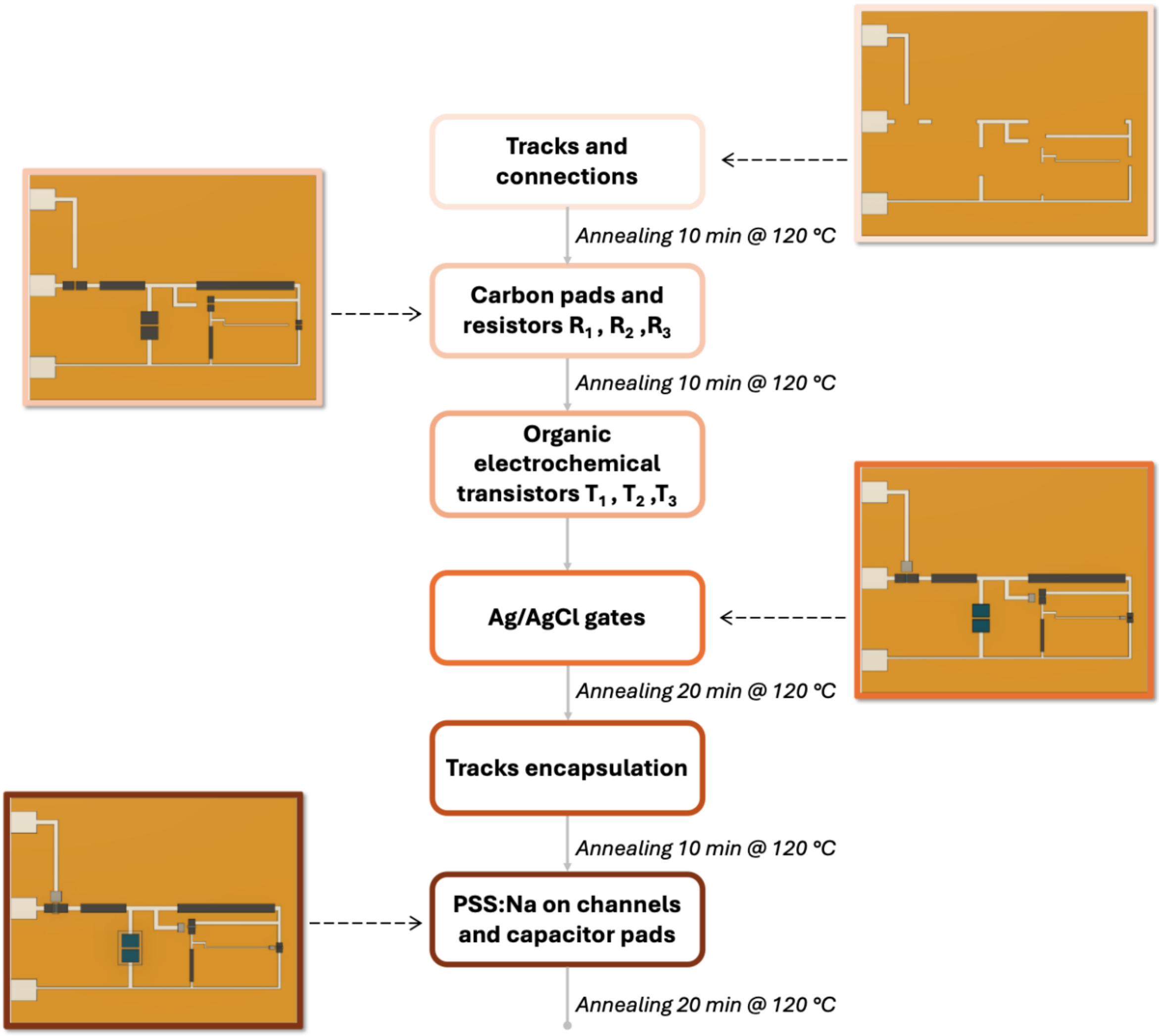

Supporting Figure S1 | Fabrication flow of a monolithically printed organic artificial neuron (OAN). Sequential micro-dispensing steps: (1) silver ink defines the electrical interconnects and contact pads; (2) carbon ink patterns the organic electrochemical transistor (OECT) source/drain electrodes, resistors, and capacitor electrodes; (3) poly(3,4-ethylenedioxythiophene) doped with poly(styrene sulfonate) PEDOT:PSS co-formulated with diethylenetriamine (PEDOT:PSS:DETA) semiconductor inks are deposited between the carbon pads to form the OECT channels, and on the capacitor electrodes to form the volumetric capacitance; (4) Ag/AgCl ink defines the OECT gates; (5) a dielectric ink encapsulates all conductive tracks; (6) polystyrene sulfonate sodium (PSS:Na) polyelectrolyte is deposited on each OECT channel and on the capacitor electrodes to form the solid-state ion reservoirs. Each layer is annealed at 120 °C for 10–20 min before deposition of the next. Ink compositions and printing parameters are detailed in the Experimental Section and Supporting Table S5, respectively.

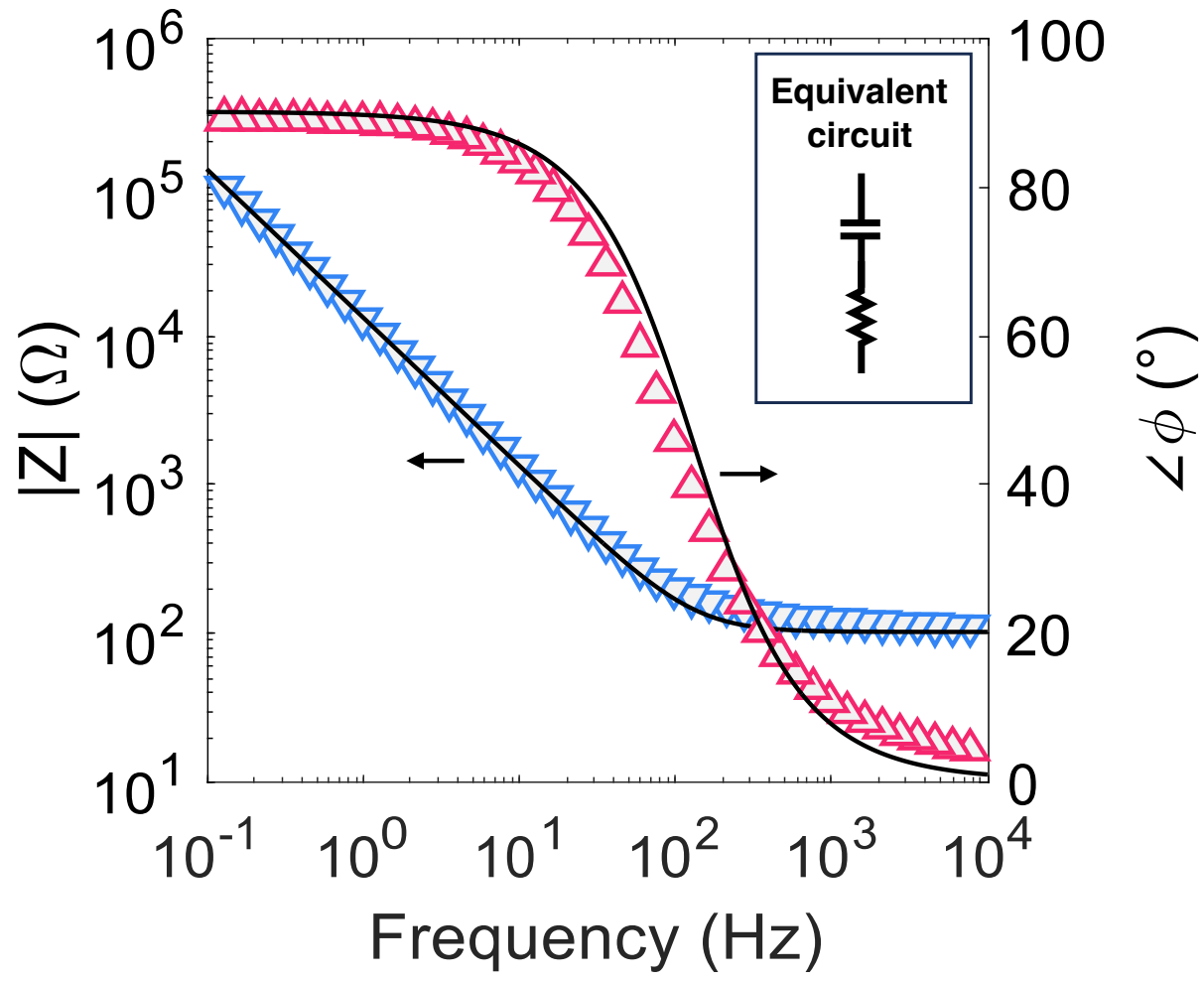

Supporting Figure S2 | Electrochemical impedance spectroscopy (EIS) of the load capacitor C_L. Measured (symbols) and modelled (solid lines) impedance magnitude (blue, left axis) and phase (pink, right axis) of C_L hydrated with 1 M NaCl. The equivalent circuit used for modelling the EIS measurements is shown in the inset, yielding R = 100 Ω and C = 12 μF.

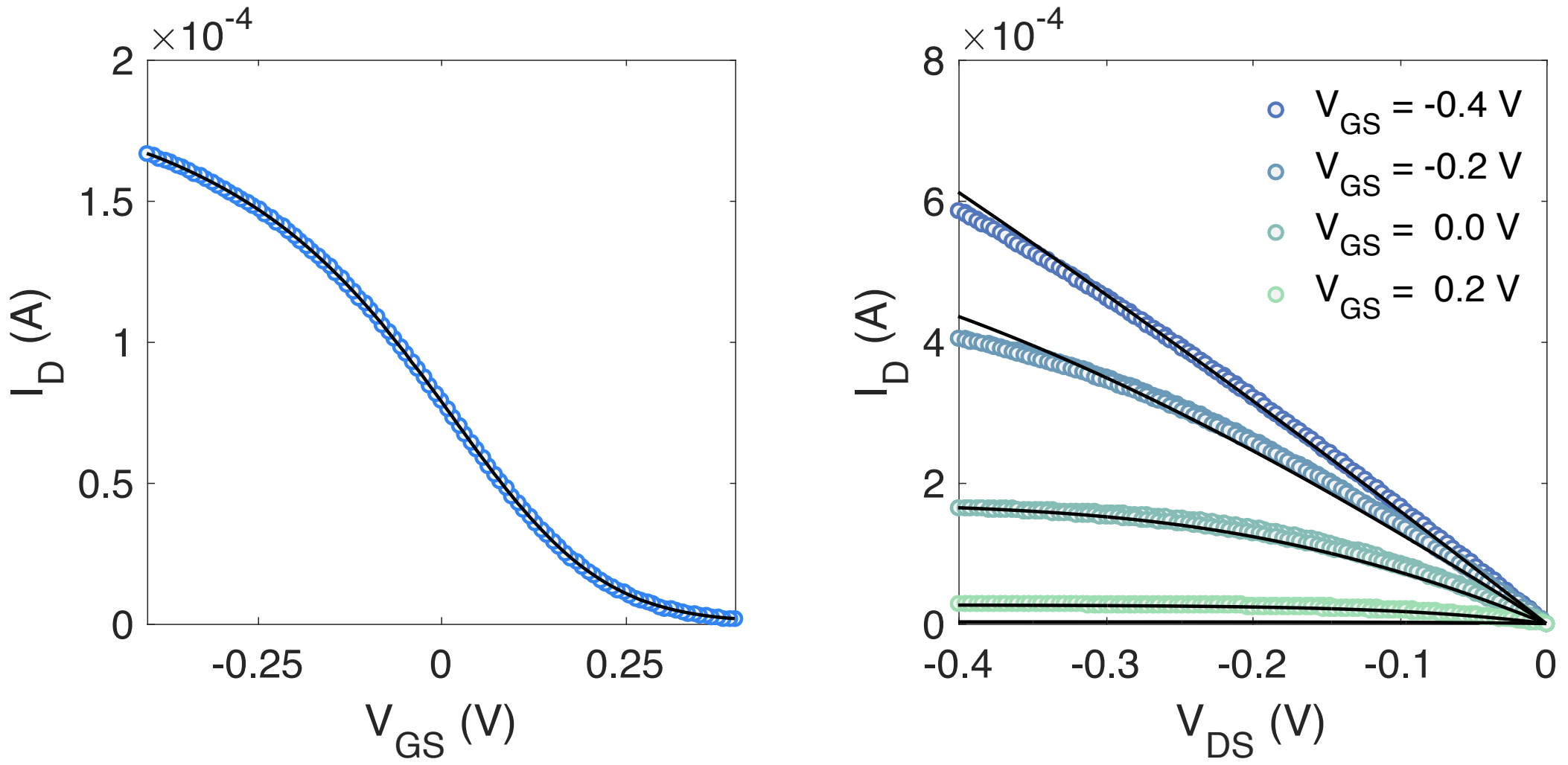

Supporting Figure S3 | Electrical characteristics of the depletion-mode OECT T_1 (PEDOT:PSS). Measured (symbols) and modelled (solid lines) transfer characteristic (V_{DS} = −0.2 V, left) and output characteristics at varying V_{GS} of T_1. Device geometry: W = L = 200 µm, t = 200 nm. Extracted model parameters: g_m/V_{SD} = 0.66 S V^{-1}, C_V = 43 F cm^{-3}, V_{TH} = 0.188 V, λ = 0.15.

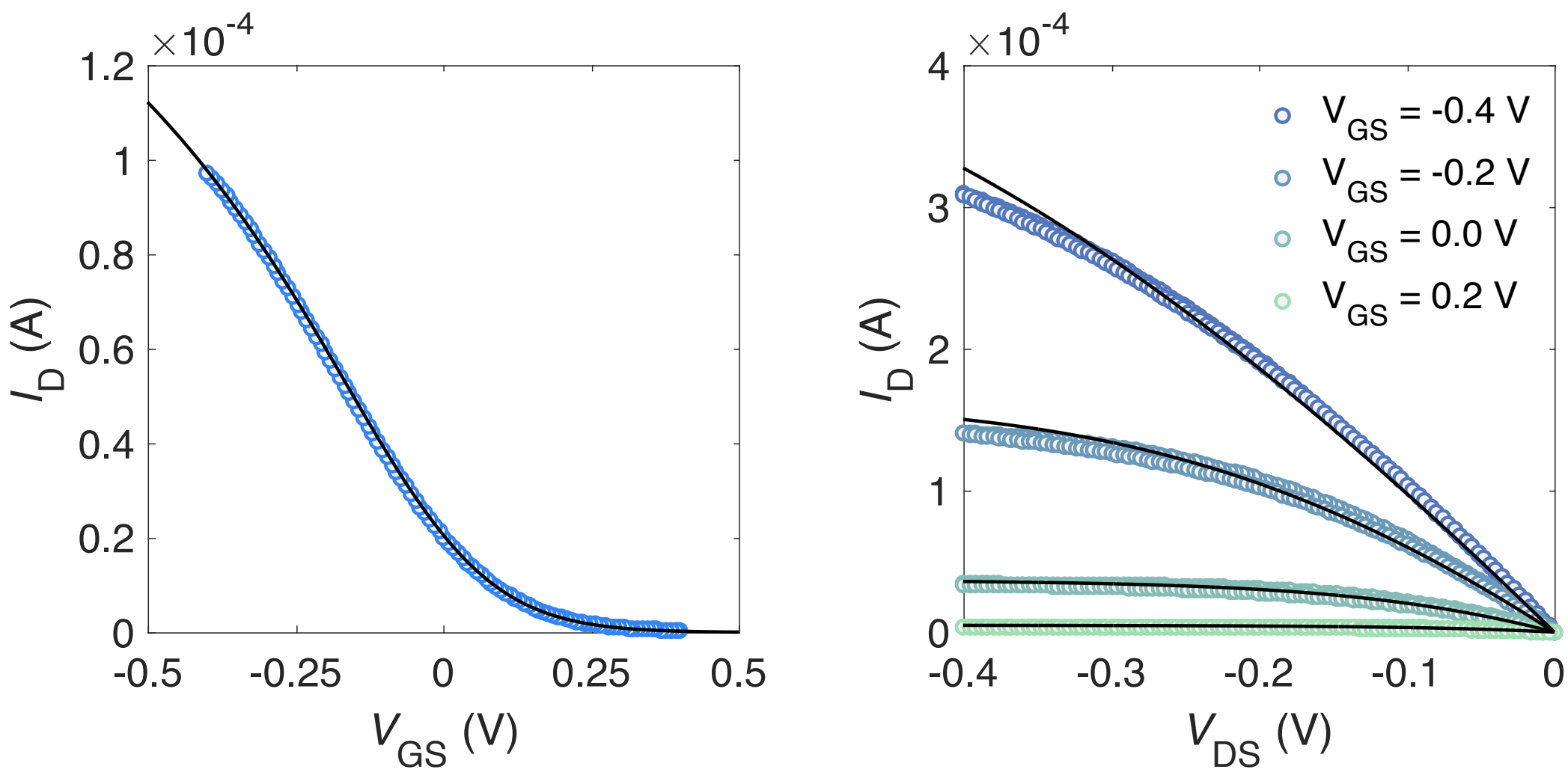

Supporting Figure S4 | Electrical characteristics of the accumulation-mode OECT T_2 (PEDOT:PSS:DETA). Measured (symbols) and modelled (solid lines) transfer characteristic ($V_{DS} = -0.2$ V, left) and output characteristics at varying V_{GS} of T_2. Device geometry: W = 130 µm, L = 50 µm, t = 200 nm. Extracted model parameters: $g_m/V_{SD} = 0.81$ S V^{-1}, $C_V = 43$ F cm^{-3}, $V_{TH} = 0.068$ V, $\lambda = 0.15$.

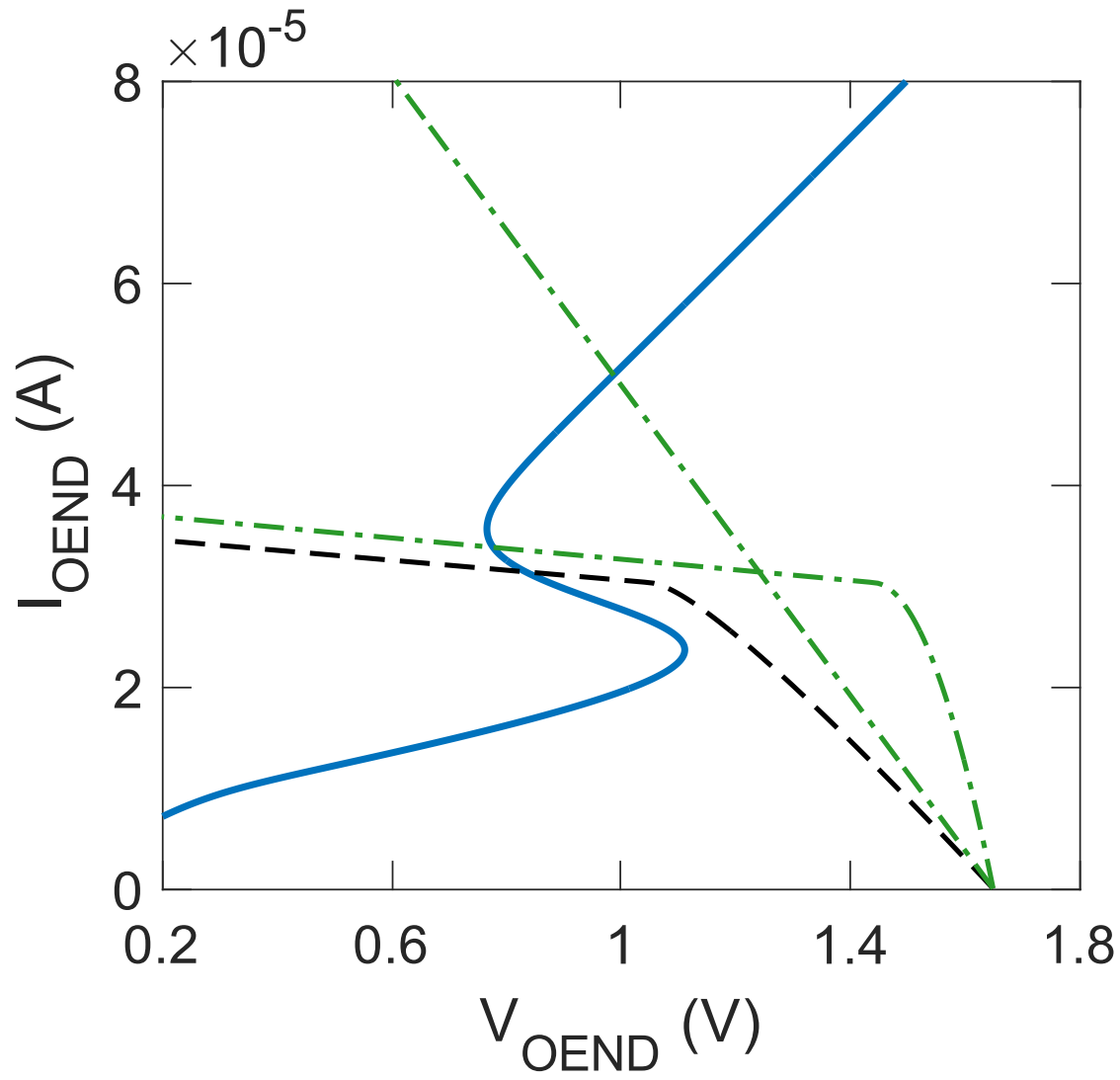

Supporting Figure S5 | Decomposition of the active load characteristic. I_{OEND}–V_{OEND} characteristic of the organic electrochemical nonlinear device (OEND) (solid blue line) and active load line (black dashed line), together with the individual contributions of R_3 alone and T_3 alone (green dash-dot lines). When only R_3 is connected, the load is linear but requires a large resistance value to ensure a single intersection with the negative differential resistance (NDR) region. When only T_3 is connected, the load follows the OECT output characteristic, providing current saturation but limited voltage control. Combining R_3 and T_3 in series yields the active load line, which simultaneously ensures a single intersection point within the NDR region and maintains biocompatible operating voltages.

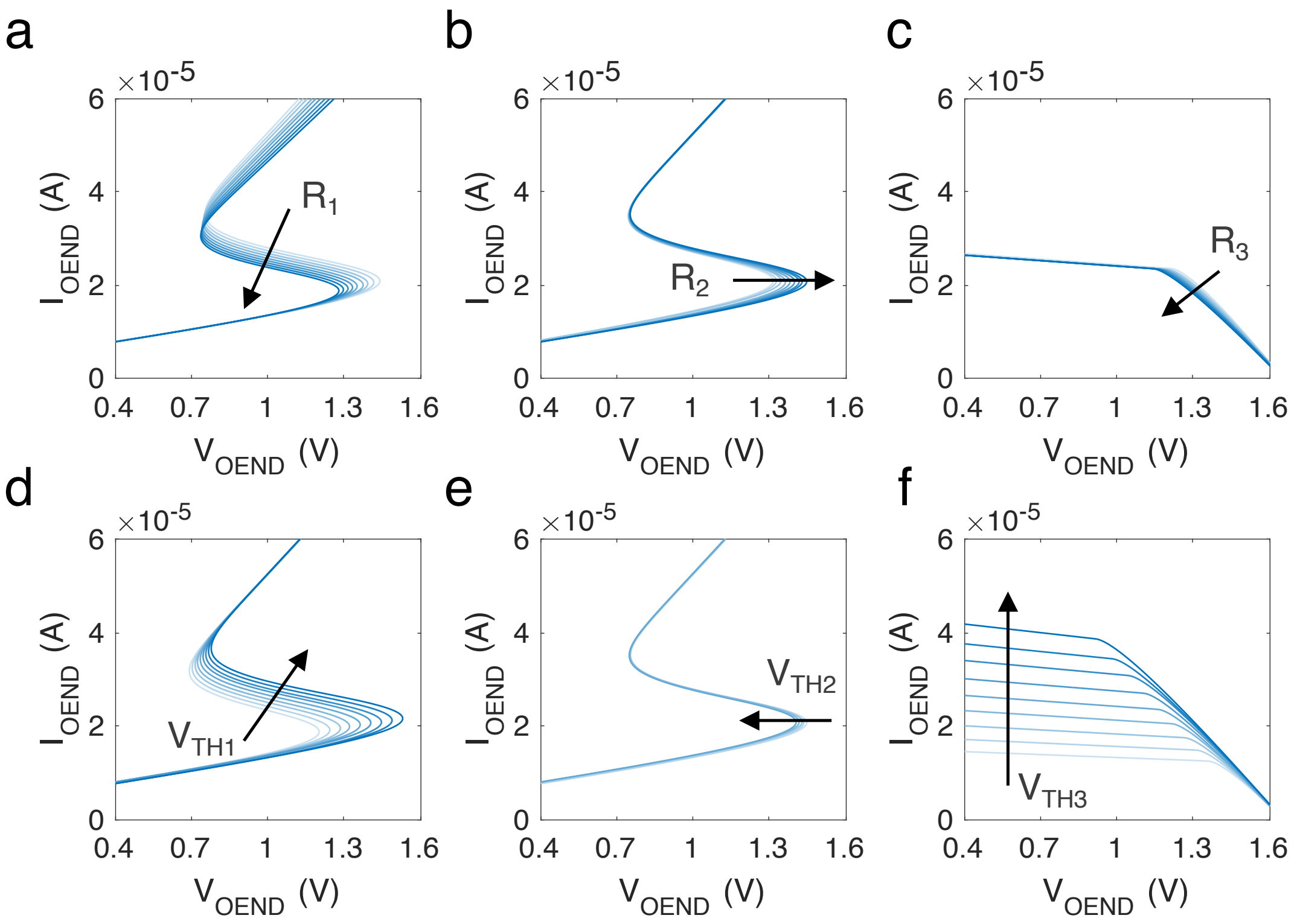

Supporting Figure S6 | Parametric sensitivity analysis of strain-dependent device parameters. Simulated I_{OEND}–V_{OEND} characteristics obtained by varying one parameter at a time. R_1, R_2, V_{TH1}, and V_{TH2} affect the intrinsic OEND S-shaped negative differential resistance (S-NDR) characteristic, whereas V_{TH3} and R_3 affect the active-load line and therefore the position of the operating point U. The arrows indicate increasing parameter values. For V_{TH1}, V_{TH2}, R_1, and R_2, the sweep ranges follow the strain-dependent values extracted in Figure 3. For V_{TH3} and R_3, a ±10% range is used. (a) R_1: 14.5–16.8 kΩ (9 linear steps). (b) R_2: 105–120 kΩ (9 linear steps). (c) R_3: 11–14 kΩ (9 linear steps). (d) V_{TH1}: 400–470 mV (9 linear steps). (e) V_{TH2}: 50–55 mV (5 linear steps). (f) V_{TH3}: 450–550 mV (9 linear steps).

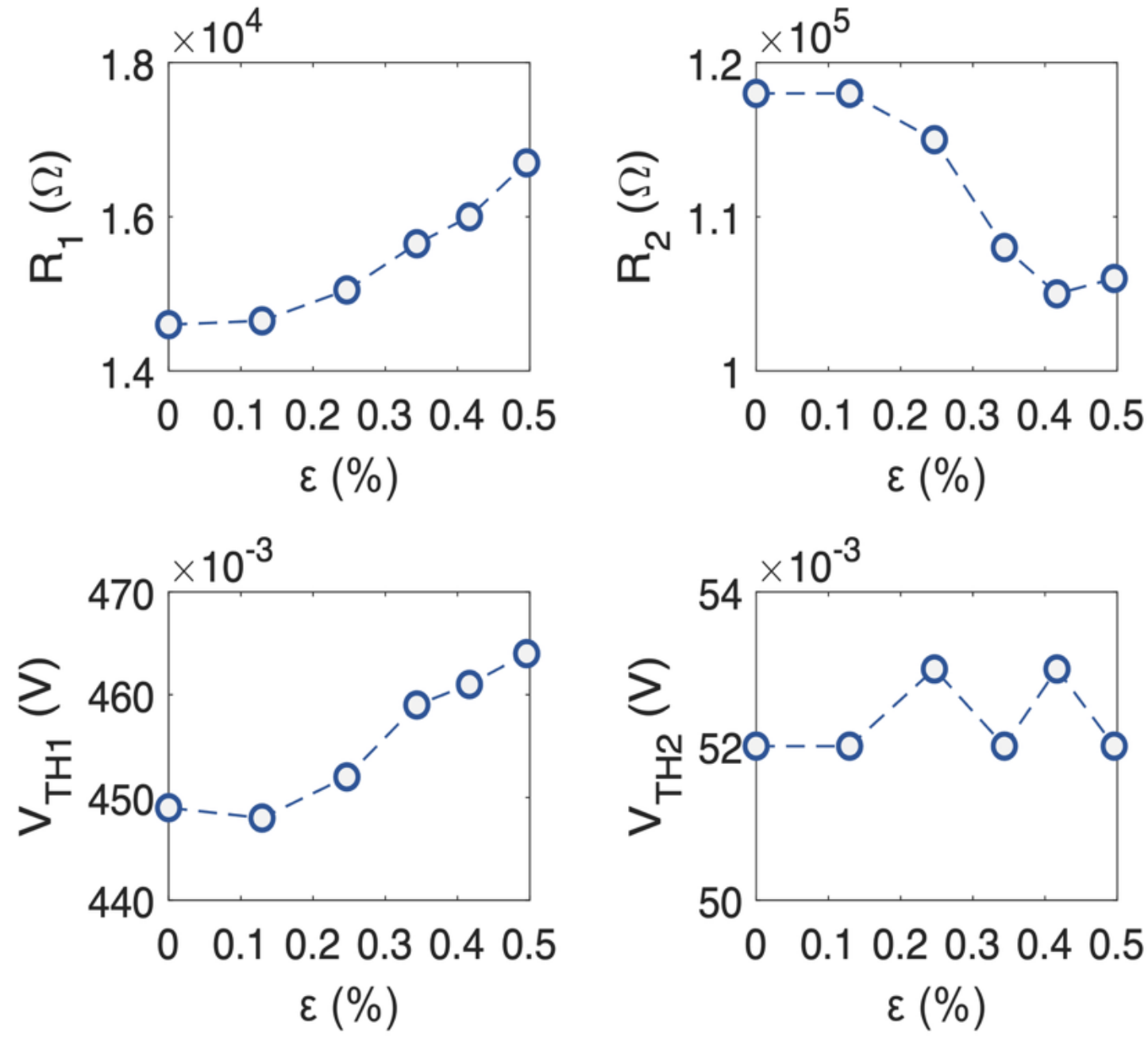

Supporting Figure S7 | Strain-dependent device parameters. Values of R_1, R_2, V_{TH1}, and V_{TH2} as a function of mechanical strain ε. R_1 increases monotonically from ~14.5 kΩ to ~17.0 kΩ, R_2 decreases from ~120 kΩ to ~105 kΩ, V_{TH1} increases from ~449 mV to ~467 mV, and V_{TH2} remains approximately constant at ~52 mV.

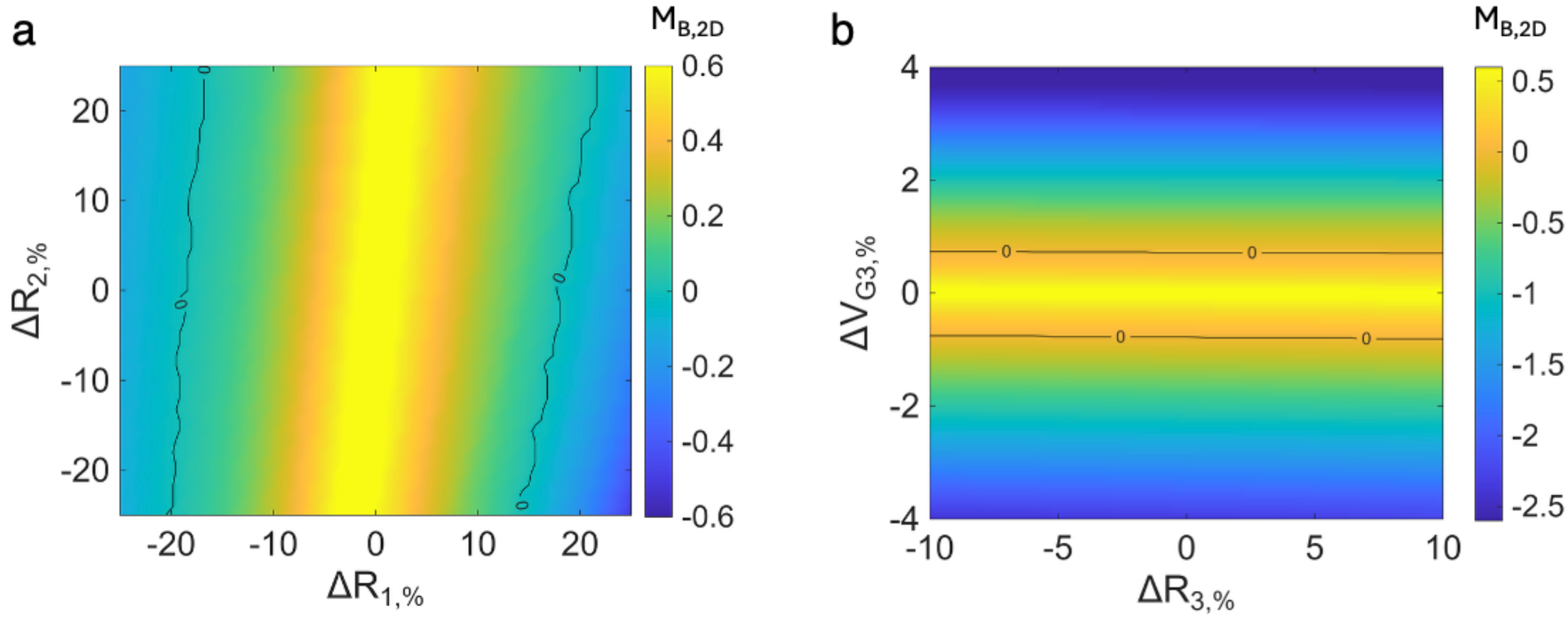

Supporting Figure S8 | Design window and tolerance analysis of the OAN. (a) Simulated R_1-R_2 design map obtained using the normalized two-dimensional NDR margin $M_{B,2D}$ (see Supporting Note 2). $M_{B,2D}$ is defined as the signed distance between the operating point U and the closest boundary point of the NDR segment in the V_{OEND}-I_{OEND} plane. Positive values indicate that U lies inside the NDR region, corresponding to oscillatory operation, whereas negative values indicate stable operation outside the NDR region. The black contour marks $M_{B,2D}$ = 0, i.e. the transition between silent and oscillatory states. The map was obtained by varying R_1 and R_2 by ±25% around their nominal values and shows an extended oscillatory region around the nominal design point, with stronger sensitivity to R_1 than to R_2. (b) Simulated R_3-V_{G3} design map obtained by varying R_3 by ±10% and V_{G3} by ±4% around their nominal values. The map shows that V_{G3} provides an effective tuning parameter to move U across the NDR boundary and compensate changes in the active-load configuration.

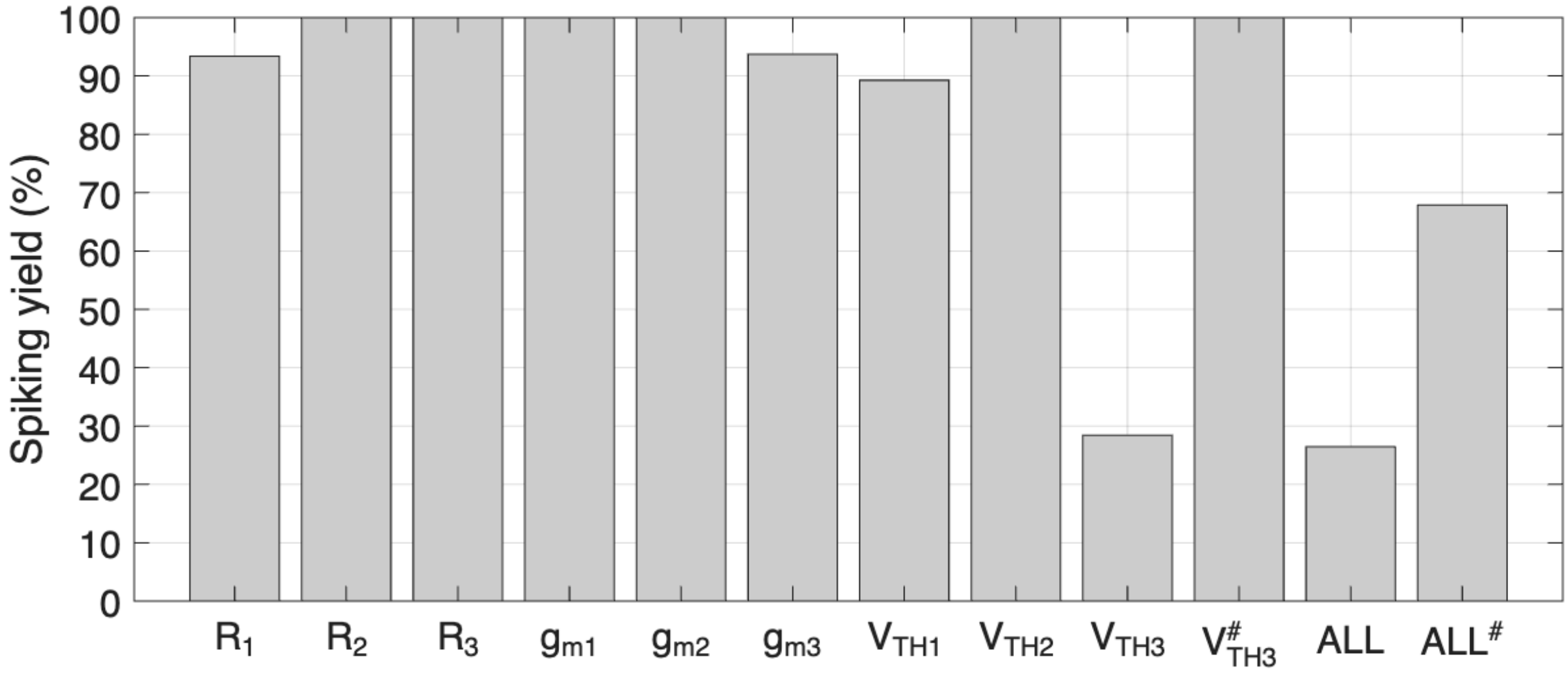

Supporting Figure S9 |. Monte Carlo tolerance analysis. Each parameter was varied according to a Gaussian distribution centered at its nominal value with standard deviation equal to 10% of the nominal value. For the single-parameter analysis, only the parameter indicated on the x-axis was stochastically varied while all others were kept at their nominal values. The spiking yields are R_1 = 93.38%, R_2 = 100%, R_3 = 100%, g_{m1} = 100%, g_{m2} = 100%, g_{m3} = 93.70%, V_{TH1} = 89.26%, V_{TH2} = 100%, and V_{TH3} = 28.43%. A total of 5000 samples was used for each single-parameter analysis. Compensation of V_{TH3} through V_{G3} restores the single-parameter V_{TH3} yield to 100% ($V_{TH3}^{\#}$ bar). When all parameters are varied simultaneously, V_{TH3} compensation increases the spiking yield from 26.47% (ALL bar – without compensation) to 67.90% ($ALL^{\#}$ bar – with compensation). In the all-parameter analysis, R_1, R_2, R_3, g_{m1}, g_{m2}, g_{m3}, V_{TH1}, V_{TH2}, and V_{TH3} were stochastically varied simultaneously using 10000 samples.

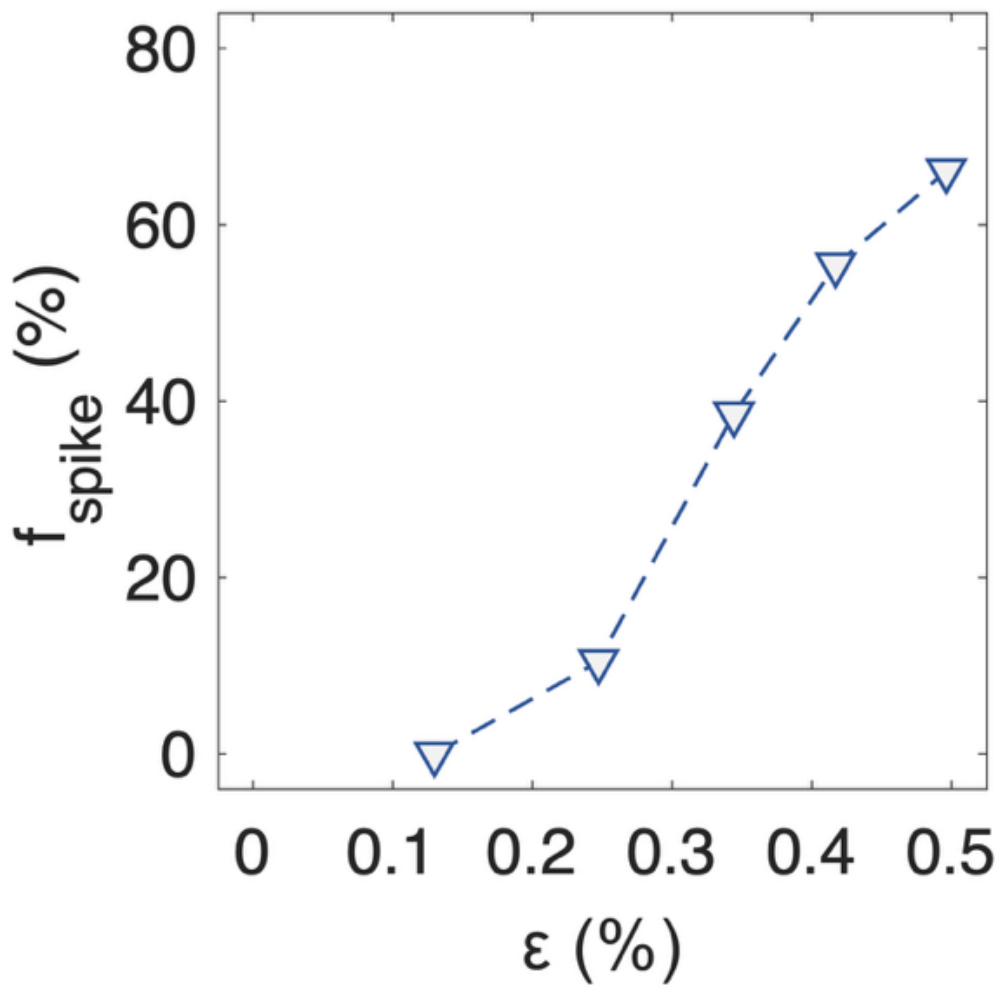

Supporting Figure S10 | Strain-dependent oscillation frequency in the mechanically induced excitation regime. Oscillation frequency f_{spike} as a percentage of its maximum value, plotted as a function of mechanical strain ε, extracted from the V_{spike} time trace of Figure 5a. f_{spike} increases monotonically from 0% at ε = 0.13% to 67% at ε = 0.50%, reflecting the progressive displacement of the operating point U deeper into the NDR region with increasing strain. Dashed line is a guide to the eye.

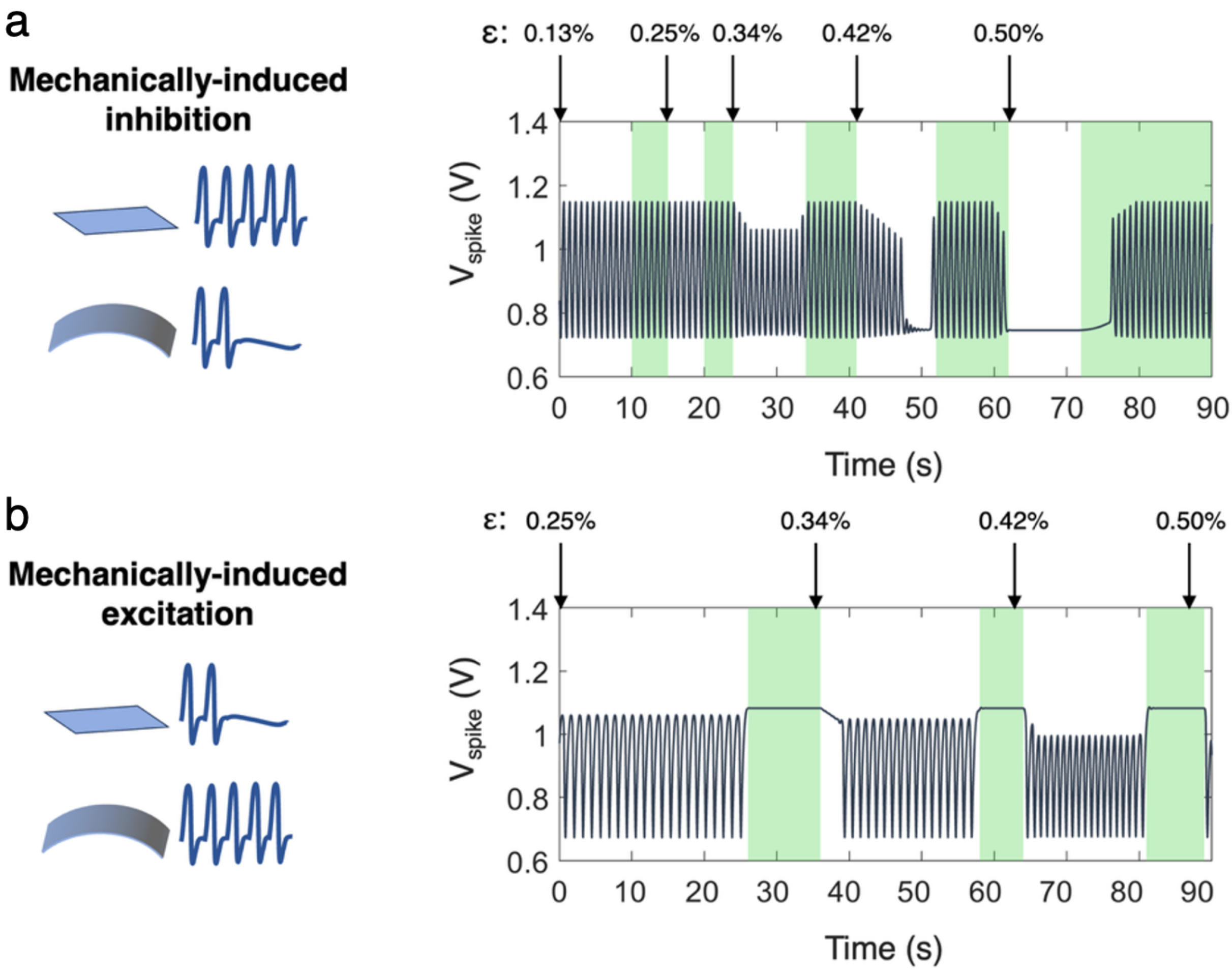

Supporting Figure S11 | Simulated spiking dynamics for mechanically induced (a) inhibition and (b) excitation. Simulated V_{spike} as a function of time under stepwise increasing mechanical strain ε, corresponding to the experimental data of Figure 4a (top, inhibition regime) and Figure 5a (bottom, excitation regime). Green regions indicate resting phases with no applied bending; arrows mark the onset of each deformation step and the corresponding asymptotic strain value. In the inhibition regime (a), the OAN is silenced above $\varepsilon \approx 0.42\%$; in the excitation regime (b), sustained bursting is triggered above $\varepsilon \approx 0.25\%$.

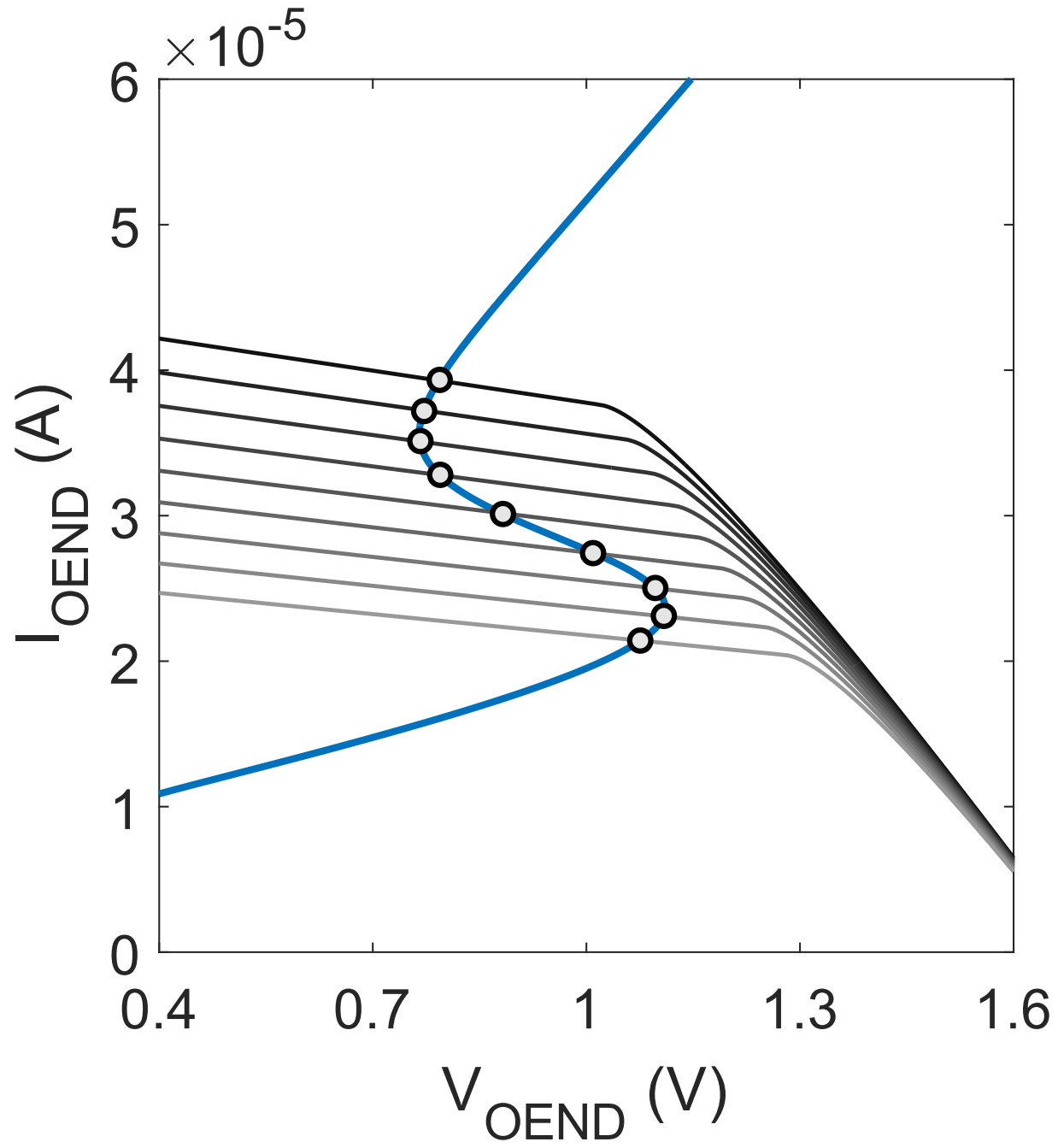

Supporting Figure S12 | Load line tuning via V_{G3}. I_{OEND}–V_{OEND} S-curve (blue) and load lines for increasing V_{G3} (gray, dark to light). This analysis isolates the effect of V_{G3}: the OEND S-NDR characteristic is kept fixed, while V_{G3} modulates the T_3 drain current and shifts the active-load line mainly vertically. Since T_3 is a depletion-mode transistor, increasing V_{G3} reduces the drain current, shifting the load line downward. At low V_{G3} (dark gray), the operating point U lies on the upper positive-slope branch of the S-curve and the OAN is silent. At intermediate V_{G3} values, U enters the NDR region and sustained oscillations are triggered. At high V_{G3} (light gray), U moves to the lower positive-slope branch and spiking is suppressed again. V_{G3} thus provides continuous and reversible control over neuronal excitability.

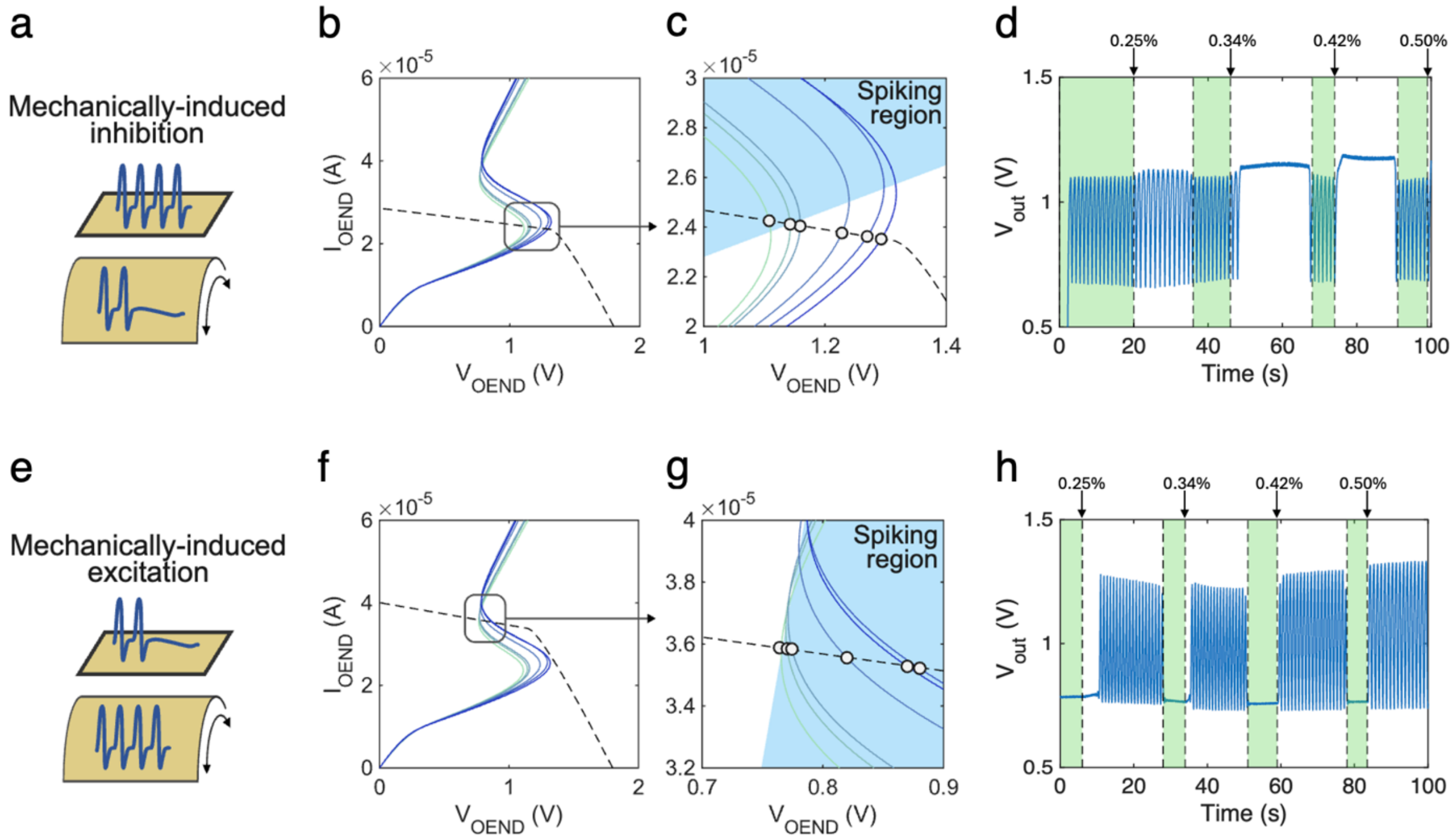

Supporting Figure S13 | Orthogonal-bending response of the OAN. (a–d) Mechanically induced inhibition under orthogonal bending. (a) Schematic of the inhibition regime under a 90°-rotated bending direction with respect to the main geometry. (b) Simulated I_{OEND}–V_{OEND} S-NDR characteristics and active-load line under increasing orthogonal strain. The curves evolve from light green to dark blue with increasing ε. (c) Zoom of the operating-point region, showing that increasing orthogonal strain moves U out of the NDR region, thereby suppressing oscillations. (d) Measured V_{spike} as a function of time under stepwise orthogonal bending, confirming mechanically induced inhibition. (e–h) Mechanically induced excitation under orthogonal bending. (e) Schematic of the excitation regime under the orthogonal bending direction. (f) Simulated I_{OEND}–V_{OEND} S-NDR characteristics and active-load line under increasing orthogonal strain. (g) Zoom of the operating-point region, showing that increasing orthogonal strain moves U into the NDR region, thereby triggering oscillations. (h) Measured V_{spike} as a function of time under stepwise orthogonal bending, confirming mechanically induced excitation. These results show that orthogonal bending reverses the direction of the S-NDR displacement compared with the main bending geometry, while inhibition and excitation remain programmable by repositioning the active-load line.

Supporting Table S1 | Current-mode OEND operating regimes

Current-mode regime	*Dominant current path*	*Internal condition*	*Differential behavior*
Low-current NDR (left) branch	R_1-T_1-R_2	T_1 ON, T_2 OFF	Positive resistance
NDR branch	R_1-T_1-R_2 and R_1-T_2	T_2 turns ON; current redistributes	Negative resistance: V_{OEND} decreases while I_{OEND} increases
High-current NDR (right) branch	R_1-T_2	T_1 OFF, T_2 ON	Positive resistance

Supporting Table S2 | Scaling considerations for compact OAN implementations.

Design element	*Role in the mechano-chemical OAN*	*Scaling considerations*
Circuit topology	Defines the OEND, active load, and capacitor connections	Preserved while reducing footprint by placing components and interconnects closer together
R_1, R_2	Strain-sensitive elements that shift the S-NDR curve	$\Delta R = R \times \varepsilon$; target resistance and strain sensitivity can be preserved through geometrical ratios, sheet resistance, thickness, material selection, or compact oriented/serpentine geometries
R_3	Sets the active-load slope together with T_3 and changes the linear-to-saturation point of T_3	Co-designed with T_3 to maintain the desired load-line intersection with the S-NDR curve
T_3/V_{G3}	Provides nonlinear active load and post-fabrication tuning	Bias tuning can compensate device-to-device and scaling-induced variations
C_L	Sets charging/discharging dynamics and spiking timescale	Changes the oscillation frequency and requires re-optimization with the OECT currents and active-load line
Flexible substrate	Transfers mechanical strain to circuit elements	Same nominal strain can be obtained at smaller footprint by adjusting substrate thickness, bending radius, and mechanical neutral plane
Layout / resistor orientation	Determines direction and magnitude of strain-induced parameter shifts	Miniaturized layouts should preserve or engineer the relative orientation between strain field and resistive elements
V_{IN}/V_{G3}	Set operating point U relative to the NDR region	Reprogrammed after scaling to set the desired excitation/inhibition windows

Supporting Table S3 | Component-level role in OAN operation.

Component / parameter	*Primary effect*	*Role in system behavior*
R_1	Positive feedback on T_1; horizontal shift of OEND S-NDR	Dominant strain-sensitive component controlling S-NDR displacement
R_2	Current level of high-conductance OEND branch	Controls current span and amplitude of the S-NDR region
T_1 / V_{TH1}	Depletion/turn-off transition	Defines one switching boundary of the S-NDR region
T_2 / V_{TH2}	Accumulation/turn-on transition	Defines the onset and asymmetry of the S-NDR region
R_3	Slope of active-load line	Determines how the load line intersects the S-NDR curve
T_3 / V_{TH3}	Nonlinear current limitation of active load	Sets current level and curvature of the load line
V_{G3}	Electrical modulation of T_3 current	Shifts load line without changing intrinsic OEND S-NDR
Ion concentration at T_3	Electrochemical modulation of V_{TH3} and $V_{G3} - V_{TH3}(c)$	Shifts load line through concentration-dependent T_3 operation
C_L	Charge-storage element	Sets transient spiking dynamics and oscillation timescale
V_{IN}	Bias voltage	Sets voltage window and load-line positioning

Supporting Table S4 | Truth table of the multimodal OAN response. M = 0 and M = 1 indicate the absence and presence of mechanical bending, respectively. C = 0 and C = 1 indicate the low and high ion concentration states, respectively.

Programmed OAN configuration	*Mechanical stimulus M*	*Chemical stimulus C*	*OAN state*	*Physical interpretation*
Mechanical excitation & chemical inhibition	0	0	Silent	U outside the NDR region
	0	1	Silent	Chemical input shifts the T_3–R_3 active-load line and keeps U out of the NDR region
	1	0	Spiking	Bending shifts the OEND S-NDR curve and moves U into the NDR region
	1	1	Silent	Chemical input shifts the T_3–R_3 active-load line and moves U out of the NDR region
Mechanical inhibition & chemical excitation	0	0	Spiking	U inside the NDR region
	0	1	Spiking	Chemical input shifts the T_3–R_3 active-load line and keeps U in the NDR region
	1	0	Silent	Bending shifts the OEND S-NDR curve and moves U out of the NDR region
	1	1	Spiking	Chemical input shifts the T_3–R_3 active-load line and moves U back into the NDR region

Supporting Table S5 | Additive manufacturing printing parameters

Ink	*Printing pressure (mbar)*	*Travel pressure (mbar)*	*Printing velocity (mm/s)*	*Needle size (µm)*
Silver	3000	2900	0.25	50
Carbon	3500	3000	0.25	50
Ag/AgCl	1500	700	0.75	100
PEDOT-A	300	100	0.45	20
PEDOT-D	100	30	0.45	20
PSS:Na	100	30	0.45	20
Encapsulant	1500	500	1	100

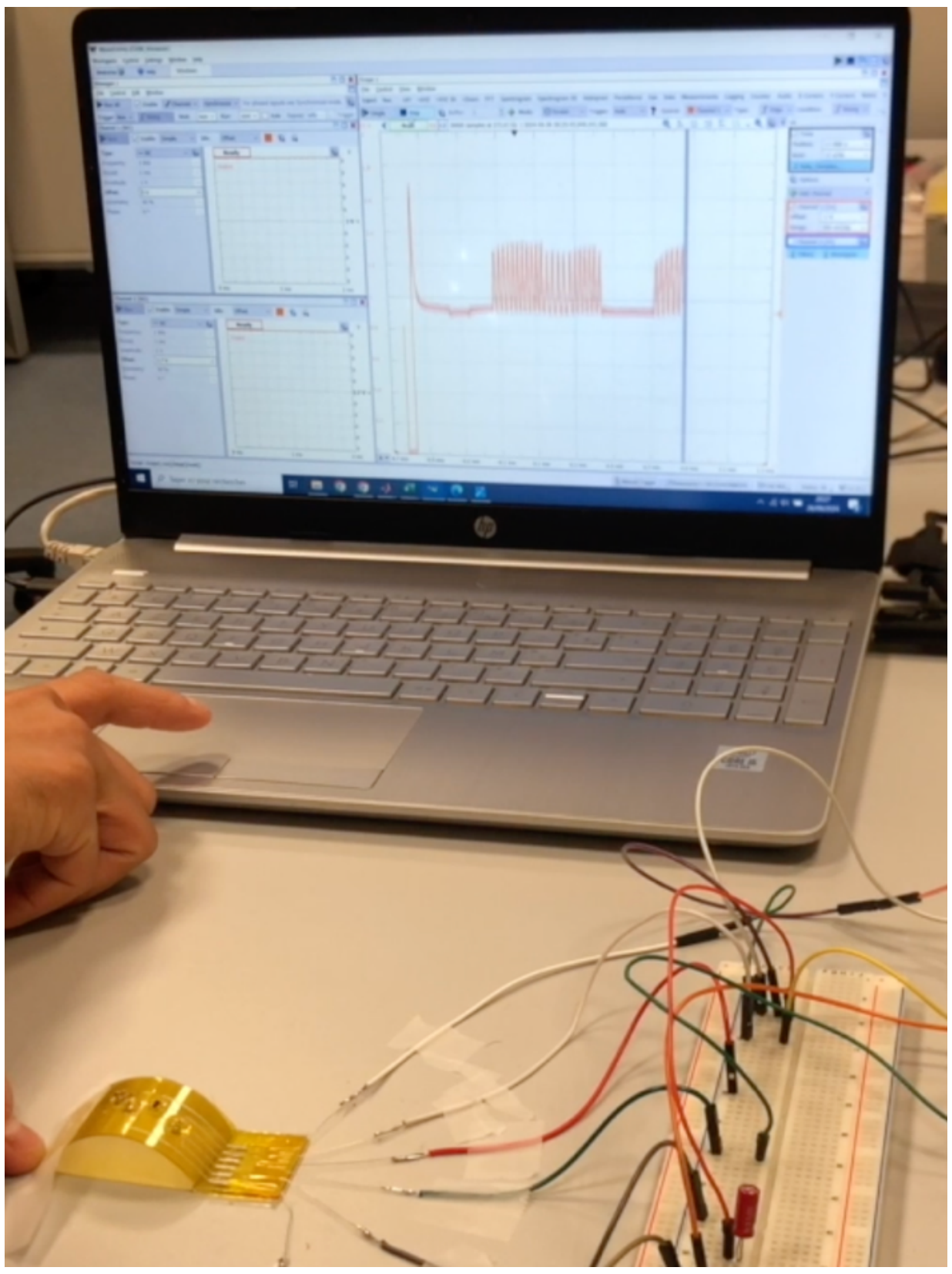

Supporting Video 1 | Mechano-electrochemical response of the OAN.

Supporting Note 1 | OECT drain current model

The Bernads-Malliaras model has been used to describe the OECT drain-current. The energy disorder as proposed by Friedlein et al.,[1,2] the channel length modulation as reported by Torricelli et al., [3] and the ion concentration-dependent threshold voltage as demonstrated by Romele et al.[4] have been included. The resulting model reads:

$$I_{\mathrm{D}} = \frac{\Gamma}{\gamma} V_{\mathrm{P}}^{2-\gamma} [\|V_{\mathrm{S}} - V_{\mathrm{G}} + V_{\mathrm{T}}\|^{\gamma} - \|V_{\mathrm{D}} - V_{\mathrm{G}} + V_{\mathrm{T}}\|^{\gamma}](1 + \lambda V_{SD}) \qquad (1)$$

where

$$\Gamma = \frac{W}{L} t\mu C_{\mathrm{v}} \qquad (2)$$

$$V_{\mathrm{P}} = \frac{q p_0}{C_{\mathrm{v}}} \qquad (3)$$

$$\gamma = \frac{E_0}{k_{\mathrm{B}} T} + 1 \qquad (4)$$

$$V_{\mathrm{TH}} = V_{\mathrm{P}} - V_{\mathrm{SH}} + V_{ISM} \qquad (5)$$

where $W, L, t,$ are the channel width, length, and thickness, respectively, μ is the hole mobility, C_{v} is the volumetric capacitance, γ is a parameter accounting for energy disorder, E_0 is the disorder parameter describing the energetic width of the tail of the density of states, λ accounts for channel length modulation, k_{B} is the Boltzmann constant, T is the temperature, V_{TH} is the threshold voltage, q is the elementary charge, p_0 is the intrinsic doping of the semiconductor, V_{SH} accounts for the voltage shift as a function of the ion concentration. The $\|\cdot\|$ operator is defined as:

$$\|x\| = x \qquad \text{if } x > 0$$

$$\|x\| = 0 \qquad \text{if } x < 0$$

According to the Supporting Equations (1)-(5), the OECT drain current depends on the applied voltages ($V_{\mathrm{S}}, V_{\mathrm{D}}, V_{\mathrm{G}}$) and on the physical and geometrical parameters ($\Gamma, V_{\mathrm{T}}, \gamma$).

(6)

The voltage V_{SH} accounts for the voltage shift as a function of the ion concentration,[4] and it reads:

$$V_{\mathrm{SH}} = V_{\mathrm{GE}} + V_{\mathrm{EP}} \tag{7}$$

$$V_{\mathrm{GE}} = \frac{k_{\mathrm{B}}T}{\mathrm{q}} \log(c) \tag{8}$$

$$V_{\mathrm{EP}} = \frac{k_{\mathrm{B}}T}{\mathrm{q}} \operatorname{asinh}\left(\frac{z_{\mathrm{fix}}N_{\mathrm{fix}}}{2zc}\right) \tag{9}$$

where c is the ion concentration in the electrolyte, z and z_{fix} are the valence of the ions dissolved into the electrolyte and the valence of fixed charge into the polyelectrolyte, respectively, and N_{fix} is the fixed charges in the bulk of the polyelectrolyte. The factor V_{SH} (Supporting Equation (7)) includes the contributions of the Nernst's potential V_{GE} (Supporting Equation (8)) at the non-polarizable gate electrode/electrolyte interface and the Donnan equilibrium potential in the value V_{EP} (Supporting Equation (9)).

Supporting Note 2 | Design-window metric and Monte Carlo tolerance analysis

For each simulated parameter set, the OEND current–voltage characteristic was computed and the NDR segment was identified as the portion for which $dI_{OEND}/dV_{OEND} < 0$. The two boundary points of this segment were denoted as $B^- = (V_{NDR}^-, I_{NDR}^-)$ and $B^+ = (V_{NDR}^+, I_{NDR}^+)$. The NDR boundary points were recalculated for each parameter combination. The operating point U = (V_U,I_U) was obtained from the intersection between the OEND characteristic and the active-load line. To quantify the design margin, we defined a normalized two-dimensional NDR margin $M_{B,2D}$ as the signed distance between U and the closest NDR boundary point:

$$M_{B,2D} = \delta(U) \times min\left[\sqrt{\left(\frac{V_U - V_{NDR}^-}{\Delta V_{NDR}}\right)^2 + \left(\frac{I_U - I_{NDR}^-}{\Delta I_{NDR}}\right)^2}, \sqrt{\left(\frac{V_U - V_{NDR}^+}{\Delta V_{NDR}}\right)^2 + \left(\frac{I_U - I_{NDR}^+}{\Delta I_{NDR}}\right)^2}\right] \tag{10}$$

where:

$$\delta(U) = \begin{cases} +1 & V_{NDR}^- \leq V_U \leq V_{NDR}^+ \\ -1 & otherwise \end{cases} \tag{11}$$

$$\Delta V_{NDR} = V_{NDR}^+ - V_{NDR}^- \tag{12}$$

$$\Delta I_{NDR} = I_{NDR}^+ - I_{NDR}^- \tag{13}$$

V_U and I_U are the voltage and current coordinate of the operating point U, respectively, V_{NDR}^+ and I_{NDR}^+ are the voltage and current coordinate of the NDR upper-limit, respectively, V_{NDR}^- and I_{NDR}^- are the voltage and current coordinate of the NDR lower-limit, respectively. Therefore, $M_{B,2D}$ accounts for both voltage and current shift. Positive values of $M_{B,2D}$ indicate that the operating point lies within the NDR region, whereas negative values indicate that it lies outside the NDR region. $M_{B,2D} = 0$ identifies the transition between oscillatory and silent operation.

Supporting References

[1] J. T. Friedlein, S. E. Shaheen, G. G. Malliaras, R. R. McLeod, "Optical Measurements Revealing Nonuniform Hole Mobility in Organic Electrochemical Transistors," *Advanced Electronic Materials* **1** (2015): 1500189, https://doi.org/10.1002/aelm.201500189

[2] D. A. Bernards, G. G. Malliaras, "Steady-State and Transient Behavior of Organic Electrochemical Transistors," *Advanced Functional Materials* **17** (2007): 3538-3544, https://doi.org/10.1002/adfm.200601239

[3] F. Torricelli, E. C. P. Smits, J. R. Meijboom, A. K. Tripathi, G. H. Gelinck, L. Colalongo, Z. M. Kovács-Vajna, D. M. de Leeuw, E. Cantatore, "Transport Physics and Device Modeling

of Zinc Oxide Thin-Film Transistors-Part II: Contact Resistance in Short Channel Devices," *IEEE Transactions on Electron Devices* **58** (2011): 3025-3033, https://doi.org/10.1109/TED.2011.2159929

[4] P. Romele, M. Ghittorelli, Z. M. Kovács-Vajna, F. Torricelli, "Ion buffering and interface charge enable high performance electronics with organic electrochemical transistors," *Nature Communications* **10** (2019): 3044, https://doi.org/10.1038/s41467-019-11073-4
